# Large Stabilization Effects by Intramolecular Beryllium Bonds in *Ortho*-Benzene Derivatives

**DOI:** 10.3390/molecules26113401

**Published:** 2021-06-04

**Authors:** Tsai I-Ting, M. Merced Montero-Campillo, Ibon Alkorta, José Elguero, Manuel Yáñez

**Affiliations:** 1 Departamento de Química, Módulo 13, Facultad de Ciencias, and Institute of Advanced Chemical Sciences (IAdChem), Universidad Autónoma de Madrid, Campus de Excelencia UAM-CSIC, Cantoblanco, 28049 Madrid, Spain; seawind1113@hotmail.com; 2 Instituto de Química Médica, IQM-CSIC, Juan de la Cierva, 3, 28006 Madrid, Spain; iqmbe17@iqm.csic.es

**Keywords:** beryllium bonds, non-covalent interactions, bonding, intramolecular interactions

## Abstract

Intramolecular interactions are shown to be key for favoring a given structure in systems with a variety of conformers. In *ortho*-substituted benzene derivatives including a beryllium moiety, beryllium bonds provide very large stabilizations with respect to non-bound conformers and enthalpy differences above one hundred kJ·mol^−1^ are found in the most favorable cases, especially if the newly formed rings are five or six-membered heterocycles. These values are in general significantly larger than hydrogen bonds in 1,2-dihidroxybenzene. Conformers stabilized by a beryllium bond exhibit the typical features of this non-covalent interaction, such as the presence of a bond critical point according to the topology of the electron density, positive Laplacian values, significant geometrical distortions and strong interaction energies between the donor and acceptor quantified by using the Natural Bond Orbital approach. An isodesmic reaction scheme is used as a tool to measure the strength of the beryllium bond in these systems in terms of isodesmic energies (analogous to binding energies), interaction energies and deformation energies. This approach shows that a huge amount of energy is spent on deforming the donor–acceptor pairs to form the new rings.

## 1. Introduction

Non-covalent interactions (NCI), first introduced by J. D. van der Waals in the late 19th century [1], occupy a well-deserved relevant place in modern chemistry, biochemistry and material science and they are responsible, for instance, for the self-assembly in supramolecular structures in living beings, the efficiency of new materials for hydrogen storage or simply the properties of water and possess important implications for life on Earth [2,3,4,5]. However, a great variety of interactions covering a considerable range of energies (from a few to more than 100 kJ·mol^−1^) are included under the NCI label. They present some common features but are in fact associated to many different elements from the periodic table acting as Lewis acceptors towards Lewis donors. The characterization of NCI has certainly enriched the concept of chemical bonding initially associated, in a seminal work by Linus Pauling [6], to electron sharing, which is an idea that will be expanded by including, in addition to conventional covalent or ionic bonds, new weak interactions such as the hydrogen bonds [7,8]. Nowadays, there is a long list of NCIs according to the acceptor nature [9]: the aforementioned hydrogen bonds (H) [10], dihydrogen bonds (H) [11,12], alkaline-earth bonds (atoms of column 2 of the periodic table) [13,14], triel bonds (column 13) [15,16], tetrel bonds (column 14) [17], pnictogen bonds (N, P, As) [18,19], chalcogen bonds (O, S, Se, Te) [20] or halogen bonds (F, Cl, Br, I) [21,22].

Beryllium bonds, which are a type of alkaline-earth bond, are stabilized by electrostatic forces in combination with a certain amount of charge transfer [13] such as common hydrogen bonds. The electron-deficient nature of beryllium leads to strong interactions with typical Lewis bases holding lone pairs such as nitrogen or oxygen-containing bases, but also unexpectedly with boron acting as a Lewis base and it demonstrates the versatility and excellent capabilities of beryllium as a Lewis acid [23]. When a donor forms a beryllium bond with a given BeX_2_ moiety, the *p* empty Be orbital and the σ* antibonding Be-X orbital are involved in the closed-shell interaction accepting charge, with some consequences on the BeX_2_ geometry such as the elongation of the Be-X bonds and the bending of the X-Be-X linear arrangement. Deformation is, therefore, a clear signature of the strength of this kind of bond [24]. The charge redistribution upon beryllium bond formation provokes great changes on the intrinsic physicochemical properties of the system acting as a Lewis base to the point that strong acids can behave as bases and vice versa [25,26].

Taking into account the ability of a beryllium bond to dramatically change the nature of the donor system, it is reasonable to think of beryllium bonds as powerful modulators of the intramolecular stability of a system containing a potential Lewis pair involving beryllium [27]. Intramolecular magnesium bonds, a related alkaline-earth NCI, have been shown to play an important role on malonaldehyde-like systems [28]. Similarly, intramolecular hydrogen bonds (IMHBs) are considered responsible for the stability of the most stable conformer in 1,2-dihidroxybenzene (catechol) based on several experimental [29,30,31,32] and theoretical studies [33,34,35], although the real existence of such IMHB has been questioned by some authors [36]. What is true is that *ortho*-substituted benzene derivatives, thanks to its structural simplicity and a certain rigidity, can be an excellent model for testing the stability given by a non-covalent intramolecular interaction between the substituents at positions 1 and 2 and that allows a systematic modification of the *ortho* substituents without further modifications of the hosting molecular framework.

**Scheme 1:**
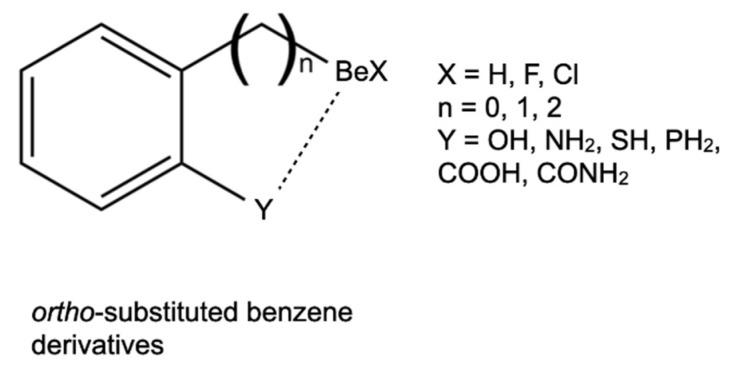
*Ortho*-substituted benzene derivatives studied including a Be moiety as a Lewis acid that is capable of forming intramolecular beryllium bonds.

Following this idea, in this work we intend to evaluate the importance that a beryllium bond may have on *ortho*-substituted benzenes (see Scheme 1) in which a set of substituents containing beryllium (-BeH, -BeF, and -BeCl, within carbon chains of different length), acting as Lewis acids, is attached to position 1 and a set of substituents containing donors of different types (Y = OH, NH_2_, SH, PH_2_, COOH, and CONH_2_), acting as Lewis bases, is attached to position 2. Our goal is to analyze the effects that the intramolecular beryllium bonds formed between the basic sites of the Y substituent and the Be atom (dotted line in Scheme 1) will have on the stabilization of the corresponding *ortho*-substituted benzene and the origin of these effects. It should be noted that for substituents COOH and CONH_2_ and the two different binding sites O or N are available to form the beryllium bond. The compounds have been selected based on their potential scientific interest although they are not known experimentally.

## 2. Computational Details

All the structures were fully optimized at the M06-2X/6-31+G(d,p) level of theory, with a posterior refinement of the energies with a larger 6-311+G(d,p) basis set. This density functional is well suited for the description of non-covalent interactions [37] and the chosen basis set is a good compromise between computational cost and accuracy given the size of the largest systems considered in this work. An ultrafine grid was used for all these DFT calculations. The Gaussian16 program [38] was used to obtain the different stationary points, which were characterized as the minima of the potential energy surface by computing the harmonic vibrational frequencies. Enthalpies are obtained by adding the M06-2X/6-31+G(d,p) thermal enthalpy correction to the electronic energy at the M06-2X/6-311+G(d,p) level.

The topology of the electron density was studied using the AIMAll program [39] within the Quantum Theory of Atoms in Molecules (QTAIM) framework [40]. This analysis identifies the points of topological interest of chemical relevance of different kinds: bond critical points (BCPs), ring critical points (RCP), cage critical points (CCP) and nuclear attractors (NA). Among these points, we are particularly interested in the ρ values at the BCPs as they are good indicators of the strength of a given bond embedded in different chemical frameworks. The Laplacian of the electron density (∇^2^ρ) and the total energy density (H) are a reflection of the nature of the bond. For a beryllium bond, we would expect positive Laplacian densities and energy densities around zero or slightly below zero. We have also used the NCI (Non-Covalent interaction) index [41] to characterize the intramolecular interactions observed in one of the structures. This method is based on the reduced density gradient and allows us to visualize, in the real space, strongly attractive, strongly repulsive and weakly attractive or repulsive non-covalent interactions. For an easier interpretation, we only used the 3D-plots with a simple color code in this work where blue, red and green stand for strongly attractive, strongly repulsive and van der Waals-range interactions, respectively. The natural bond orbital (NBO) analysis [42], which is based on the generation of localized hybrid orbitals corresponding to Lewis-type representations of the molecular structures, permits the calculation of second-order orbital interaction to quantitatively characterize donations and backdonations among occupied and empty localized molecular orbitals involved in intermolecular and/or intramolecular interactions.

**Scheme 2:**
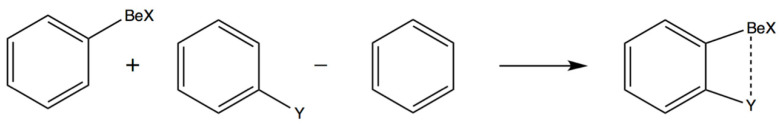
Isodesmic reactions used to estimate the intramolecular interaction energy due to the beryllium bonds exemplified for the particular case of carbon chains with *n* = 0 (see Scheme 1).

Common methods to estimate the strength of intramolecular bonding are changes in geometrical parameters and topological descriptors. The estimation of the energy involved in the intramolecular interaction is also useful but admits more than one definition. The relative stability between conformers, depending on the presence or absence of the interaction, is one of the definitions. The electronic energy associated to an isodesmic reaction is another tool for calculating intramolecular interaction energies [43,44,45,46,47] and, in particular, intramolecular hydrogen bonds [48,49,50,51,52,53]. An intramolecular interaction may be formally built up by a fictitious chemical reaction and the energy of this reaction is representative of the interaction energy. The energies for the isodesmic reactions were computed according to the energy difference between products and reactants shown in Scheme 2.

Once the isodesmic energies (E_iso_) are obtained, we can further refine our analysis by decomposing this energy into two terms, the deformation energy (E_def_) and the interaction energy (E_int_) such that E_iso_ = E_int_ + E_def_, in a similar manner as was proposed in the previous literature to analyze reaction rates [54]. The interaction energy is the result of subtracting the energy of the reactants from the energy of the product in Scheme 2 but with the geometry they present in the product. Hence, the distorted geometry of the first reactant is obtained by replacing the Y substituent of the product by a H atom. For the second reactant, it is the BeX substituent (the one replaced by H) and for the third reactant both substituents are replaced by H. The deformation energy is just the difference between the energy of the isodesmic reaction and the aforementioned defined interaction energy.

## 3. Results and Discussion

In this section, we firstly discuss the energy differences between conformers to estimate the stabilization caused by the formation of beryllium bonds and the trends observed according to the substitution patterns. The observed trends are then rationalized in a second step by means of the NBO decomposition scheme and the topological analysis of the beryllium bond. Finally, the isodesmic energies and the decomposition of these energies into deformation and interaction energies will help to fully understand the stabilization provided by the different substituents.

### 3.1. Stabilization Gained by Beryllium Bond Formation

A total of 108 conformers were obtained at the M06-2X/6-311+G(d,p)//M06-2X/6-31+G(d,p) level (see Table S1 in the Supporting Information for more details). The structures will be labelled, if it is required, by simply writing the interacting pair Y:(CH_2_)_n_BeX (donor–acceptor; example: NH_2_:BeCl).

**Figure 1:**
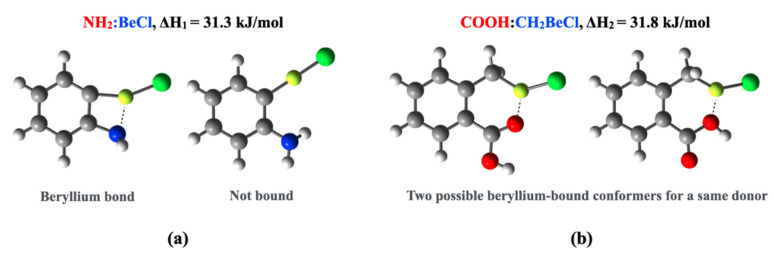
(**a**) Meaning of enthalpy ΔH_1_ taking NH_2_:BeCl as a suitable example. Enthalpy ΔH_1_ gives the stabilization provided by the formation of a beryllium bond. (**b**) Meaning of enthalpy ΔH_2_ taking COOH:CH_2_BeCl as a suitable example. Enthalpy ΔH_2_ gives the energy difference depending on the binding site for a same donor. The color code for the atoms is the following: hydrogen: white; carbon: grey; nitrogen: blue; oxygen: red; chlorine: green; and beryllium: yellow.

The stabilization energy gained by the formation of the intramolecular beryllium bond for donors Y = (OH, SH, NH_2_, PH_2_) is reported in Table 1 as the enthalpy difference ΔH_1_ between the conformer exhibiting a beryllium bond and the most stable one in which this interaction is not present (see Figure 1a for the particular case NH_2_:BeCl). For donors Y = (COOH, CONH_2_), there are two conformers stabilized by a beryllium bond depending on the binding site (O from carbonyl group or the OH/NH_2_ groups) and the stability difference between them is reported in Table 1 as enthalpy ΔH_2_ (see Figure 1b for the particular case COOH:CH_2_BeCl).

From the ΔH_1_ values in Table 1 it can be seen that the stabilization provided by the beryllium bond goes from 2.2 kJ·mol^−1^ (OH:BeH) to 87.0 kJ·mol^−1^ (NH_2_:C_2_H_4_BeCl), where in all cases the global minimum corresponds to the conformer stabilized by an intramolecular beryllium bond. Furthermore, for the set of compounds in which Y = COOH and CONH_2_, the global minimum and the second most stable conformer correspond to conformers stabilized by intramolecular beryllium bonds. Any other non-bound conformers for these donors, where found, is situated much higher in terms of energy (see Table S2). Our results show that in all cases the most stable of the two conformers is that in which the carbonyl oxygen is involved.

**Figure 2:**
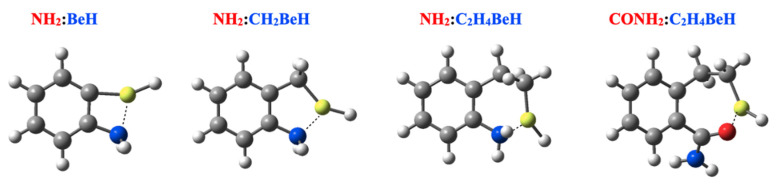
Suitable examples of the global minima where the formation of the intramolecular beryllium bond leads to the formation of an extra four-(NH_2_:BeH), five-(NH_2_:CH_2_BeH), six-(NH_2_:C_2_H_4_BeH) or seven-(CONH_2_:C_2_H_4_BeH) membered heterocycle. The atoms color code is the same as in Figure 1.

It is also important to note, as illustrated in Figure 2, that the appearance of the beryllium bond between the basic site of the Y substituent and the Be atom implies the formation of new four-membered, five-membered, six-membered or seven-membered heterocycles, depending in the length of the –(CH_2_)_n_ (*n* = 0, 1, 2) carbon chain to which the BeX is attached.

This is a crucial feature because if we look in detail at the values in Table 1 for the donors OH, SH, NH_2_ and PH_2_, it is clear that the length of the carbon chain from zero to two carbon atoms in the acceptor (BeX, CH_2_BeX, and C_2_H_4_BeX) has a huge effect on the stabilization energies since this chain determines the size of the new heterocyclic ring formed on the BeB conformer. Four membered-rings (acceptor BeX), where formed, provide small stabilization with respect to the non-bound conformer, while five-membered (CH_2_BeX) and six-membered (C_2_H_4_BeX) rings are largely stabilized in comparison with the open form. This is, at the moment, something already expected. However, it is interesting to note that when looking at the effect of X on the beryllium unit, the stabilization gained by replacing H by F is pretty similar in many cases or even lower for F. One would expect that a more electronegative atom would make Be a better acceptor in almost all situations. Results with a BeCl moiety are pretty similar to the BeF ones in the case of four-membered rings but stabilization energies are significantly larger for five-membered and six-membered rings. The highest reported value is 87.0 kJ·mol^−1^ for the NH_2_:C_2_H_4_BeCl pair. The effect of the halogen would later be clarified in the posterior sections.

It is also worth noting that, as in all Lewis pair interactions, the quality of the electron donor plays a crucial role. It is easy to appreciate that interactions of CH_2_BeX and C_2_H_4_BeX acceptors with first-row donors such as OH and NH_2_ give place to larger stabilizations than when compared to second-row donors SH and PH_2_, where the stabilization decreases in the order NH_2_ > OH > SH > PH_2_. The size effect O/S and N/P is something already observed in regular intermolecular beryllium bonds [13].

The geometrical parameters in Table 2 point in the same direction and indicates strong stabilizations by the formation of the beryllium bonds. Indeed, the C-Be-X angle, which in the absence of interaction is strictly linear, becomes significantly distorted, especially for X = Cl as a sign of the strength of the interactions. It is clear, for instance, that the newly formed four-membered rings present high deviations of this angle from 180° for OH and NH_2_ strong donors, while much modest deviations are found for SH or PH_2_. Interestingly, when the length of the carbon chain increases favoring the formation of five-membered and six-membered rings, the C-Be-X angles are closer to 120° than to 180° in all cases, indicating that we can properly speak of tri-coordinated beryllium atoms.

**Figure 3:**
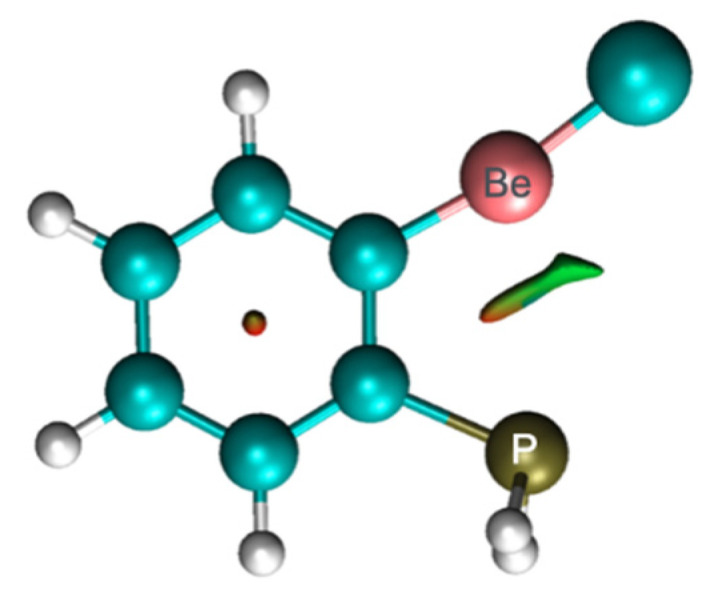
NCI plot for the PH_2_:BeCl structure showing the weak interaction between the donor and acceptor (green-colored isosurface) and a slightly red-colored region denoting a certain connection between the four atoms susceptible of forming a ring through a BeB.

The BeX acceptor series (the formation of a four-member ring) deserves some additional discussion. Only NH_2_, which is the best donor in general terms, displays significant stabilizations for the formation of a four-member ring through a beryllium bond. Very large donor-Be distances and very small deformations of the C-Be-X angle (see Table 2) are found along with the relatively low stabilization energies of the BeX series. Interestingly, for the pairs interacting at long distances, a soft atom, such as S, gives rise to slightly larger stabilizations than O, regardless of whether we take BeH (2.2 vs. 12.5 kJ·mol^−1^), BeF (6.7 vs. 11.3 kJ·mol^−1^) or BeCl (7.1 vs. 11.7 kJ·mol^−1^). In other words, for four-member rings, the rigidity of the benzene framework does not allow the substituents to reach significant interactions and only an extremely good donor such as N in NH_2_ gives rise to considerable stabilizations (NH_2_:BeCl, 31.3 kJ·mol^−1^, the case illustrated in Figure 1a). This is also consistent with the fact that for these compounds no BCP associated with the Be-Y interaction is found (see electron densities at BCP in Table S3). Furthermore, coherently, for this set the second-order NBO interaction energies are about one order of magnitude smaller than those found for the remaining derivatives (see Table S4 of the Supporting Information). Hence, we conclude that the SH and PH_2_ values seem to be a reflection of the dispersion forces involved more than a reflection of the beryllium bonds themselves. In other words, the absence of a BCP between the Be atoms and the basic site in these compounds does not mean that no interaction is taking place between the donor and acceptor. For regions of the 3D-space in which the electron density is deformed by weak non-covalent interactions, but not enough to give rise to a BCP, the NCI plots (see Computational Details) are very useful. Figure 3 illustrates the particular case of the PH_2_:BeCl structure in which a green isosurface denotes an interaction within the van der Waals range between P and Be, as is suggested when looking at the stabilization energies found for these compounds (1.4 kJ·mol^−1^, Table 1). However, the reason is still not obvious as to why -OH results are similar (or even smaller) than the results for -SH or -PH_2_ in the four-membered ring cases. We will later observe that the energy analysis might provide some answers in this respect.

**Figure 4:**
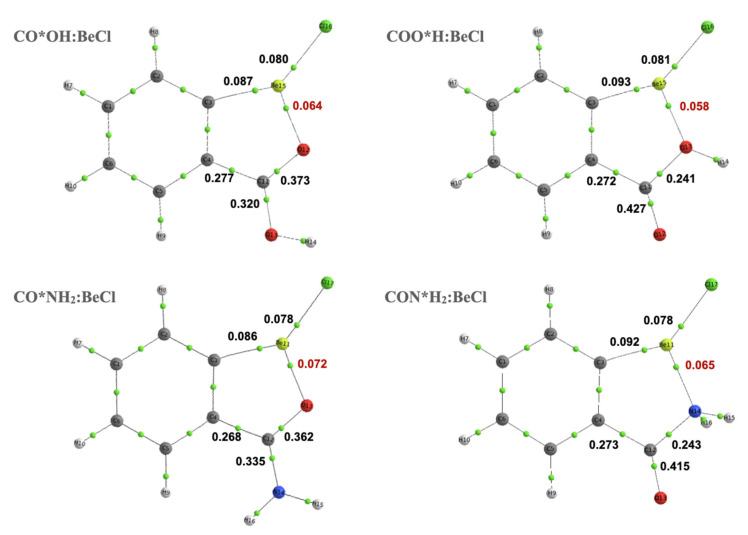
Molecular graphs for CO*OH:BeCl, COO*H:BeCl, CO*NH_2_:BeCl and CON*H_2_:BeCl derivatives showing the electron density (au) (red color) at the intramolecular beryllium bond.

The last two rows of Table 1, as mentioned above, permitted the conclusion that for Y = COOH and CONH_2_, the intramolecular beryllium bond involving the carbonyl group is systematically more stabilizing than the one involving the hydroxyl or the amino group, up to a maximum of 30.6 kJ·mol^−1^ and 57.6 kJ·mol^−1^, respectively. This is coherent with the relative basicity of the lone pairs in the COOH and CONH_2_ groups; although, in general, the isolated N is a better donor than O and resonance with the carbonyl group makes NH_2_ more available to donate its pair towards carbonyl than OH. These basicity differences are very well reflected in the corresponding molecular graphs. In Figure 4 we show, as a suitable example, a comparison between CO*OH:BeCl, COO*H:BeCl, CO*NH_2_:BeCl and CON*H_2_:BeCl. It can be observed that when the interaction takes place with the carbonyl group, the electron density at the BCP is larger for CONH_2_ (0.072 au) than for COOH (0.064 au). The role of the carbonyl group as good donors for the formation of hydrogen bonds is very well known since the 1970s and 1980s for acids, esters and amides [55,56,57,58,59]. Moreover, when the interactions involve the hydroxyl (0.058 au) or the amino group (0.065 au), they are weaker than those involving the carbonyl group but the interaction involving the amino is stronger than the interaction involving the hydroxyl group.

This picture is also consistent with the values obtained for the NBO second-order perturbation energies (see Table S4 of the Supporting Information), where CO*NH_2_:BeCl is 15 kJ·mol^−1^ larger than CO*OH:BeCl. Similarly, they are 51 kJ·mol^−1^ and 37 kJ·mol^−1^ larger for CO*OH:BeCl and CO*NH_2_:BeCl than for COO*H:BeCl and CON*H_2_:BeCl, respectively.

**Figure 5:**
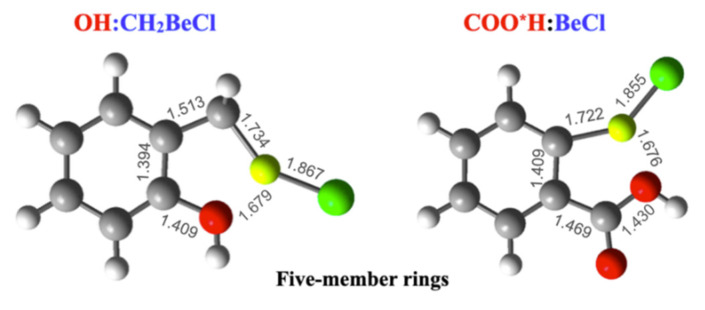
Comparison between different kinds of five-member rings depending on the donor (OH and COO^*^H). The same applies for larger rings. Distances are provided in Å.

We previously showed in Figure 2 that the extra rings formed by the beryllium bonds may contain five members, six members and seven members in total. It is important, however, to keep in mind that a direct comparison between some of the five-member or six-member rings formed could be tricky (see Figure 5 as a suitable example). For the second subset of donors, COOH and CONH_2_, the ΔH_1_ values (not shown in Table 1, see Table S2), i.e., the differences between the conformers stabilized by an intramolecular beryllium bond and those that do not present this feature can be very high for six-member rings and reaches differences larger than one hundred kJ·mol^−1^ and thus showing the much larger nucleophilicity of the oxygen atom in the carbonyl group than that of the isolated OH group. Different kinds of five member and six member rings will be compared in the following section from the topological point of view.

### 3.2. Nature of the Intramolecular Beryllium Bond

**Figure 6:**
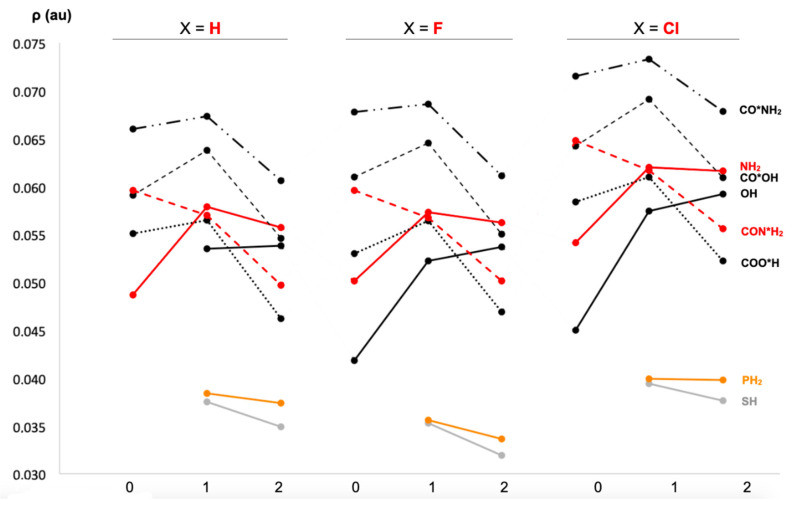
Electron density values (au) according to substitution patterns for *ortho*-substituted benzenes. The number of (CH_2_) groups in the different systems is indicated in the X axis.

The electron density at the bond critical point (BCP) allows the comparison of the same kind of beryllium bond (O-Be, N-Be, P-Be and S-Be) in different structures. Figure 6, along with Figure S1 and Table S3 in the Supporting Information, summarizes the results for the electron density of the studied beryllium bonds culminated with the collection of molecular graphs.

The electron density ρ in Figure 6 shows at a glance the effect of the carbon chain and X at the beryllium acceptor (CH_2_)_n_BeX (*n* = 0, 1, 2; X = H, F, Cl) as well as the contrast between the different kinds of oxygens (black lines) and nitrogens (red lines) as donors. Firstly, it is important to note, as mentioned above, that no BCP is found for OH:BeX (X=H), SH:BeX and PH_2_:BeX (*n* = 0) derivatives, in agreement with the very low stabilization effects due essentially to van der Waals interactions found for this subset. It can be also observed that for donors OH, SH, NH_2_ and PH_2_, beryllium bonds forming five-member and six-member rings exhibit larger electron densities than four-member rings. For donors COOH and CONH_2_, five-member and six-member rings also have larger electron densities than seven-member rings. This is a well-known effect of geometry that is already described in proton-transfer processes in pyrazoles assisted by water where the optimum number is three and if a fourth water molecule is added the effect is worsened [60]. We confirm that Cl gives place to the highest values for the electron density; the strongest beryllium bond being that of CO*NH_2_:CH_2_BeCl. Regarding the Laplacian of the density, the values are positive, which is in agreement with typical ionic or closed shell interactions, and total energy densities are around zero or eventually slightly negative.

**Figure 7:**
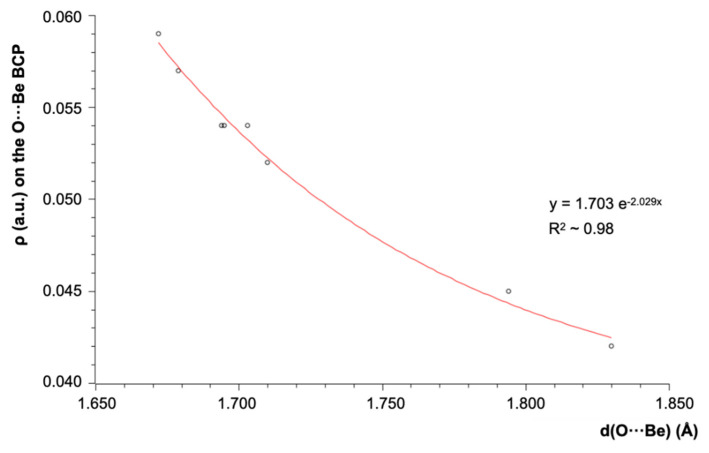
Exponential correlation between the Be-O distance and the density values at the bond critical point for the whole -OH series.

Typical strong beryllium bonds used to present [61] electron density values that correlate really well with the interatomic Y-Be distances according to an exponential fit (see Figure 7). This is the case we observe here for the strongest interactions (R^2^_OH_ = 0.98 and R^2^_NH2_ = 0.98), while poorer donors exhibit poorer correlations (R^2^_PH2_ = 0.72 and R^2^_SH_ = 0.78). Interactions through the carbonyl oxygen for both COOH and CONH_2_ groups present significantly shorter distances and a moderate correlation compared to larger beryllium bonds (R^2^_CO*_ = 0.77), likely reflecting the stabilizing effects of the cyclization process. All correlation plots are shown in Figure S2.

**Figure 8:**
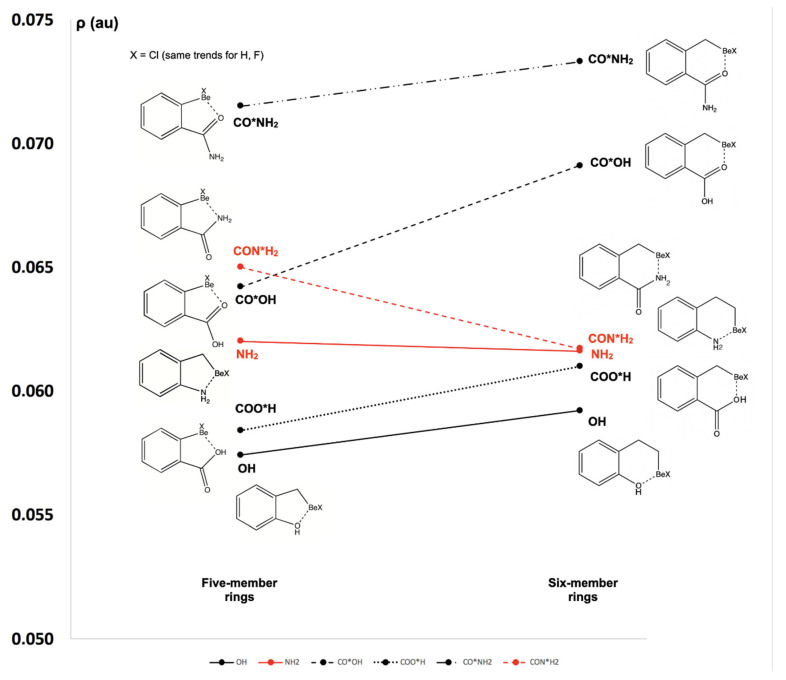
Comparison of density values for the pairs between (CH_2_)_n_BeX, where X = Cl and O, N donors for the formation of five-member and six-member rings of different kinds (see Figure 2).

For an easier comparison between O and N donor abilities and the influence of the sp^2^/sp^3^ character (which might be tricky, as pointed out before in Figure 5), in Figure 8 we focused on the effect on the beryllium bond electron density of these groups looking only at five-member and six-member rings of different kinds. As a suitable example, we selected (CH_2_)_n_BeCl substituents as acceptors. In this comparison, the ability of O as a donor increases in the order OH < COO*H < CO*OH < CO*NH_2_ and N is a better donor in the case of the amide group, although the difference between NH_2_ and CON*H_2_ is almost negligible on forming six-member rings. This might lead to the apparent conclusion that the sp^2^ character of lone pairs in carboxylic and amide groups makes them better donors towards beryllium.

### 3.3. Isodesmic Reactions

The NBO second-order interaction energies can only be used, strictly speaking, in relative terms. In order to have an energetic viewpoint on the intramolecular beryllium bonds that are investigated, we decided to use isodesmic reactions (see Scheme 2) which can provide some clues about the interaction between the donor and acceptor from a different perspective. Taking into account the conformational richness of the largest systems, we have analyzed the trends for the simplest *n* = 0 case (BeX as acceptor) for all donors since these are expected to present the lowest deformation energy values. In other words, the isodesmic energy (E_iso_), as explained in the Computational details section, is decomposed in contributions arising from the interaction (E_int_) and the deformation (E_def_) of the reactants. Table 3 summarizes all these terms depending on the different donor–acceptor pairs.

Looking at the E_int_ column, we can immediately observe that the interaction energies increase in the order PH_2_ < SH < < OH < NH_2_ for the same BeX acceptor, which is in agreement with what we observed from the electron density results and the NBO second-order energies. This is not exactly the same trend observed when only looking at the stabilization energies in Table 1 because of the very low values obtained for OH. The explanation is also clear by looking at the values of the deformation energies, which are larger for OH than for NH_2_ and much larger than for SH or PH_2_, leading to isodesmic reactions for this donor that is smaller than the ones obtained for the other three since it was the also case for the stabilization energies.

Looking now to the CONH_2_ and COOH donors at the bottom of Table 3, we can also observe, for the same BeX acceptor, larger values for the carbonyl group in the former than in the latter, which is in agreement with a better nucleophilic character of the carbonyl oxygen in amides and that was already discussed in previous sections.

A second interesting trend is the acceptor ability of beryllium on the series BeH, BeF, BeCl. If we look at the E_int_ values (i.e., no deformation taken into account), we now find that interactions are always stronger along the H, F, Cl series and this is a trend that, again, is not exactly the same and is found neither in the stabilization energies (see Table 1) nor in the E_iso_ values, demonstrating again the role of the E_def_ term. In summary, although obtained differently, the interaction energy values extracted from the isodesmic scheme are in line with the second-order energies of the NBO method and the electron densities at the BCP, while the isodesmic energies are similar to the stabilization energies associated with the formation of the intramolecular beryllium bond in Table 1.

## 4. Conclusions

Intramolecular beryllium bonds are able to strongly stabilize 1,2-benzene derivatives, in particular, when forming five-membered or six-membered rings. In those cases, simple nitrogen (-NH_2_) and oxygen (-OH) donors give place to stabilization energies above 60 kJ·mol^−1^ and up to almost 90 kJ·mol^−1^ with respect to not bound conformers. The beryllium bonds have been characterized by the existence of a bond critical point of the electron density with positive Laplacian values and total energy density values around zero. Carbonyl oxygen and amide oxygen can give place to even larger electron density values. The acceptor ability of beryllium is increased when replacing hydrogen by halogen atoms. Interaction energies obtained with NBO and an isodesmic reaction scheme indicate that the intramolecular beryllium bond itself is very strong but a significant amount of energy is used in deforming the structure to fit into the newly formed ring. This is something that is particularly clear when looking at the angle formed between beryllium and its substituents, which is far from linearity (180°).

## Figures and Tables

**Table 1:** M06-2X/6-311+G(d,p)//M06-2X/6-31+G(d,p) relative enthalpies ΔH_1_ (kJ·mol^−1^) between the beryllium bound conformer and the most stable non-bound conformer, taking the beryllium bound conformer as zero. At the bottom of the table and for substituents COOH and CONH_2_, the enthalpy difference, ΔH_2_ (kJ·mol^−1^), between the two possible conformers exhibiting a beryllium bond, the one involving the carbonyl group (systematically the most stable) and the one involving the OH (NH_2_) group, is given.

**ΔH_1_**	**Acceptor**	**BeH**	**CH_2_BeH**	**C_2_H_4_BeH**	**BeF**	**CH_2_BeF**	**C_2_H_4_BeF**	**BeCl**	**CH_2_BeCl**	**C_2_H_4_BeCl**
	Donor									
	OH	2.2	60.4	61.8	6.7	59.9	62.5	7.1	67.6	71.0
	SH	12.5	41.8	35.8	11.3	40.9	32.5	11.7	47.8	39.8
	NH_2_	23.1	69.6	74.5	26.8	73.6	74.3	31.3	81.0	87.0
	PH_2_	4.4	21.8	26.4	1.6	20.2	21.9	1.4	26.3	29.9
**ΔH_2_**	**Acceptor**	**BeH**	**CH_2_BeH**	**C_2_H_4_BeH**	**BeF**	**CH_2_BeF**	**C_2_H_4_BeF**	**BeCl**	**CH_2_BeCl**	**C_2_H_4_BeCl**
	Donor									
	COOH	14.8	28.0	25.2	19.0	30.6	24.7	18.8	31.8	27.9
	CONH_2_	51.3	53.9	50.5	56.2	57.6	54.1	54.1	57.2	56.4

**Table 2:** Be-Y distances (Å, Y atom donor of the Lewis base) and C-Be-X angle (italic characters) for the *ortho*-substituted benzenes considered in Scheme 1. Stars on COOH and CONH_2_ donors indicate which donor atom is involved in the interaction.

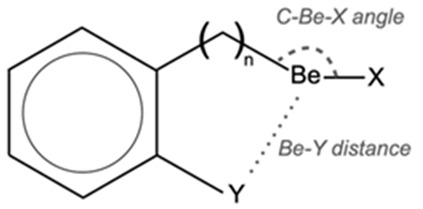									
	**BeH**	**CH_2_BeH**	**C_2_H_4_BeH**	**BeF**	**CH_2_BeF**	**C_2_H_4_BeF**	**BeCl**	**CH_2_BeCl**	**C_2_H_4_BeCl**
**OH**	1.838 *159.4*	1.695 *149.1*	1.694 *146.4*	1.830 *159.7*	1.710 *149.4*	1.703 *146.8*	1.794 *157.7*	1.679 *148.0*	1.672 *144.2*
**SH**	2.727 *168.9*	2.241 *149.2*	2.246 *147.1*	2.648 *166.4*	2.290 *149.9*	2.311 *148.1*	2.487 *160.6*	2.228 *145.9*	2.232 *143.3*
**NH_2_**	1.855 *154.6*	1.787 *145.5*	1.794 *141.7*	1.847 *154.7*	1.796 *145.2*	1.796 *142.1*	1.819 *153.7*	1.769 *144.0*	1.767 *139.7*
**PH_2_**	3.007 *172.5*	2.310 *146.4*	2.280 *142.6*	2.987 *171.3*	2.365 *147.8*	2.353 *144.2*	2.984 *170.1*	2.285 *143.4*	2.266 *139.9*
CO*OH COO*H	1.697 *143.3* 1.690 *148.8*	1.650 *137.0* 1.674 *144.8*	1.690 *138.6* 1.740 *144.9*	1.694 *143.3* 1.701 *149.0*	1.652 *138.0* 1.681 *145.2*	1.693 *138.0* 1.742 *145.4*	1.675 *141.6* 1.676 *147.2*	1.631 *135.7* 1.685 *142.8*	1.663 *135.8* 1.710 *141.1*
CO*NH_2_ CON*H_2_	1.660 *141.5* 1.773 *144.4*	1.635 *136.5* 1.779 *140.4*	1.660 *135.8* 1.828 *142.2*	1.657 *141.3* 1.779 *144.5*	1.633 *136.8* 1.787 *140.7*	1.662 *135.1* 1.832 *142.3*	1.639 *139.8* 1.751 *142.7*	1.613 *140.7* 1.761 *138.4*	1.636 *133.2* 1.797 *139.3*

**Table 3:** Isodesmic energies (E_iso_), interaction energies (E_int_) and deformation energies (E_def_) for the different compounds at the M06-2X/6-311+G(d,p) level of theory. For COOH and CONH_2_ groups, the first line corresponds to O(CO) and the second line corresponds to OH/NH_2_.

Interacting Pairs		E_iso_	E_int_	E_def_
OH	BeH	−6.1	−86.6	80.5
	BeF	−9.0	−99.1	90.1
	BeCl	−10.6	−103.9	93.3
NH_2_	BeH	−49.7	−128.1	78.4
	BeF	−53.6	−143.0	89.3
	BeCl	−59.2	−148.2	89.0
SH	BeH	−13.7	−29.3	15.7
	BeF	−14.3	−37.4	23.0
	BeCl	−15.7	−53.1	37.4
PH_2_	BeH	−7.4	−11.8	4.4
	BeF	−7.9	−13.2	5.3
	BeCl	−8.4	−13.5	5.2
COOH	BeH	−82.6	−166.3	83.7
		−66.1	−130.9	64.8
	BeF	−85.1	−173.4	88.3
		−64.3	−129.7	65.4
	BeCl	−91.2	−182.7	91.5
		−70.4	−141.4	71.0
CONH_2_	BeH	−111.2	−198.1	86.9
		−59.5	−168.4	109.0
	BeF	−114.1	−206.6	92.5
		−57.9	−172.8	114.9
	BeCl	−122.2	−216.9	94.7
		−67.8	−189.8	122.0

## Data Availability

The data presented in this study are available in the article and in the Supplementary Material.

## Supplementary Materials

The following are available, Table S1: Relative enthalpies at the M06-2X/6-31+G(d,p) level of theory, Table S2: Relative enthalpies at the M06-2X/6-311+G(d,p)//M06-2X/6-31+G(d,p) level of theory for the (CH_2_)_n_BeX:COOH not bound conformers with respect to the beryllium bond-containing ones, Table S3: Electron density at the beryllium bond BCP obtained at the M06-2X/6-311+G(d,p)//M062X/6-31+G(d,p) level of theory, Table S4: Second-order interaction energies obtained within the NBO approach between donors and the Be acceptor, Table S5: Cartesian coordinates for the Y:(CH_2_)_n_BeX 1,2-benzene derivatives (*n* = 0, 1, 2; X = H, F, Cl; Y = OH, NH_2_, SH, PH_2_, COOH, CONH_2_) optimized at the M06-2X/6-31+G(d,p) level of theory, Figure S1: Molecular graphs for the beryllium-bonded complexes at the M06-2X/6-311+G(d,p) level of theory. Values for the corresponding topological parameters are detailed in Table S3, Figure S2: Correlation between bond distances and electron energy densities for different donor groups.

## Sample Availability

Samples of the compounds are not available from the authors.
