# Supplementary material for: Large Stabilization Effects by Intramolecular Beryllium Bonds in Ortho-Benzene Derivatives
Source: Molecules. 2021 Jun 4;26(11):3401. doi: 10.3390/molecules26113401 (PMC8199991; doi:10.3390/molecules26113401)
Supplement: Supplementary file 1 [file molecules-26-03401-s001.zip › molecules-1240141-supplementary.pdf]

molecules

Article

Large Stabilization Effects by Intramolecular Beryllium Bonds in *Ortho*-Benzene Derivatives

Tsai I-Ting [1], M. Merced Montero-Campillo [1,*] Ibon Alkorta [2,*], José Elguero [2] and Manuel Yáñez [1,*]

[1] Departamento de Química, Módulo 13, Facultad de Ciencias, and Institute of Advanced Chemical Sciences (IadChem), Universidad Autónoma de Madrid, Campus de Excelencia UAM-CSIC, Cantoblanco, 28049 Madrid, Spain; seawind1113@hotmail.com
[2] Instituto de Química Médica, IQM-CSIC, Juan de la Cierva, 3. 28006 Madrid, Spain; iqmbe17@iqm.csic.es
* Correspondence: mm.montero@uam.es (M.M.M.-C.); ibon@iqm.csic.es (I.A.); manuel.yanez@uam.es (M.Y.)

Supporting Information Contents

Table S1. Relative enthalpies at the M06-2X/6-31+G(d,p) level of theory.
Table S2. Relative enthalpies at the M06-2X/6-311+G(d,p)//M06-2X/6-31+G(d,p) level of theory for the $(CH_2)_nBeX$:COOH not bound conformers with respect to the beryllium bond-containing ones.
Table S3. Electron density at the beryllium bond BCP obtained at the M06-2X/6-311+G(d,p)//M06-2X/6-31+G(d,p) level of theory.
Table S4. Second-order interaction energies obtained within the NBO approach between donors and the Be acceptor.
Figure S1. Molecular graphs for the beryllium-bonded complexes at the M06-2X/6-311+G(d,p) level of theory. Values for the corresponding topological parameters are detailed in Table S3.
Figure S2. Correlation between bond distances and electron energy densities for different donor groups.
Table S5. Cartesian coordinates for the Y:$(CH_2)_nBeX$ 1,2-benzene derivatives (n = 0, 1, 2; X = H, F, Cl; Y = OH, NH_2, SH, PH_2, COOH, $CONH_2$) optimized at the M06-2X/6-31+G(d,p) level of theory.

Table S1. M06-2X/6-31+G(d,p) relative enthalpies ΔH_1 (kJ·mol^{-1}) between the beryllium bound conformer and the most stable non-bound conformer, taking the beryllium bound conformer as zero. Relative energies ΔH_2 (kJ·mol^{-1}) are shown in second place for substituents COOH and $CONH_2$, representing the difference in energy depending on the binding site for a same donor (oxygen from carbonyl group being the most stable one). Note that in the latter case all conformers contain a beryllium bond. Additional non-bound structures are not included. .

ΔH_1	Acceptor	BeH	CH_2BeH	C_2H_4BeH	BeF	CH_2BeF	C_2H_4BeF	BeCl	CH_2BeCl	C_2H_4BeCl
	Donor									
	**OH**	5.3	62.2	63.1	9.1	61.4	63.2	11.0	70.1	72.5
	**SH**	12.5	41.6	36.1	10.8	41.0	32.5	12.0	48.8	40.8
	**NH_2**	25.8	76.3	75.6	28.8	75.1	75.3	34.7	84.8	89.4
	**PH_2**	4.6	22.2	26.8	1.7	21.3	23.3	1.7	28.2	31.8
ΔH_2	**COOH**	19.5	31.3	27.1	23.7	33.9	27.4	24.2	36.0	31.4
	**$CONH_2$**	56.0	56.6	51.4	60.8	60.7	56.1	59.0	61.0	58.7

Table S2. Relative free energies at the M06-2X/6-311+G(d,p)//M06-2X/6-31+G(d,p) level of theory for the $(CH_2)_nBeX$:COOH not bound conformers with respect to the BeB-containing ones.

Acceptor	BeH	CH_2BeH	C_2H_4BeH	BeF	CH_2BeF	C_2H_4BeF	BeCl	CH_2BeCl	C_2H_4BeCl
**Donor**									
**COOH**	-	104.0	63.9	-	104.8	67.3	-	112.7	74.7

Table S3. Electron density at the BeB bond critical points obtained at the M06-2X/6-311+G(d,p)//M06-2X/6-31+G(d,p) level of theory.

Acceptor	BeH	CH_2BeH	C_2H_4BeH	BeF	CH_2BeF	C_2H_4BeF	BeCl	CH_2BeCl	C_2H_4BeCl
**Donor**									
**OH**	-	0.054	0.054	0.042	0.052	0.054	0.045	0.057	0.059
**SH**	-	0.038	0.035	-	0.035	0.032	-	0.039	0.038

**NH_2**	0.049	0.058	0.056	0.050	0.057	0.056	0.054	0.062	0.062
**PH_2**	-	0.038	0.037	-	0.036	0.034	-	0.040	0.040
**CO*OH**	0.059	0.064	0.055	0.061	0.065	0.055	0.064	0.069	0.061
**COO*H**	0.055	0.057	0.046	0.053	0.056	0.047	0.058	0.061	0.052
**CO*NH_2**	0.066	0.067	0.061	0.068	0.069	0.061	0.072	0.073	0.068
**CON*H_2**	0.060	0.057	0.050	0.060	0.057	0.050	0.065	0.062	0.056

Table S4. Second-order interaction energies obtained within the NBO approach between donors and the Be acceptor ($kJ \cdot mol^{-1}$) for the *ortho*-substituted benzenes summarized in Scheme **1.**

**Acceptor**	**BeH**	**CH_2BeH**	**C_2H_4BeH**	**BeF**	**CH_2BeF**	**C_2H_4BeF**	**BeCl**	**CH_2BeCl**	**C_2H_4BeCl**
**Donor**									
**OH**	57.2	488.9	442.0	85.0	503.5	530.2	100.0	564.8	575.9
**SH**	27.7	541.7	583.4	49.5	478.1	459.6	93.9	726.3	725.5
**NH_2**	72.1	472.0	440.0	128.7	497.1	479.7	132.8	542.0	522.4
**PH_2**	14.1	524.7	584.0	12.8	415.6	446.9	16.2	707.7	749.0
**CO*OH**	182.1	717.8	601.5	183.4	745.3	627.0	198.7	815.0	692.7
**COO*H**	154.1	602.4	503.3	134.9	608.6	521.2	147.4	692.7	568.1
**CO*NH_2**	196.1	741.2	672.0	201.3	782.4	676.1	213.1	839.8	770.2
**CON*H_2**	94.5	474.4	394.6	169.5	505.4	431.3	176.4	567.9	489.7

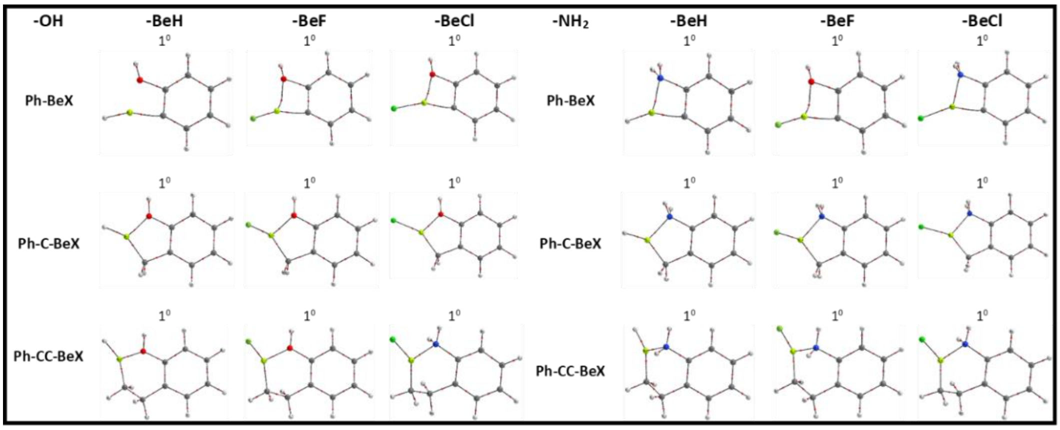

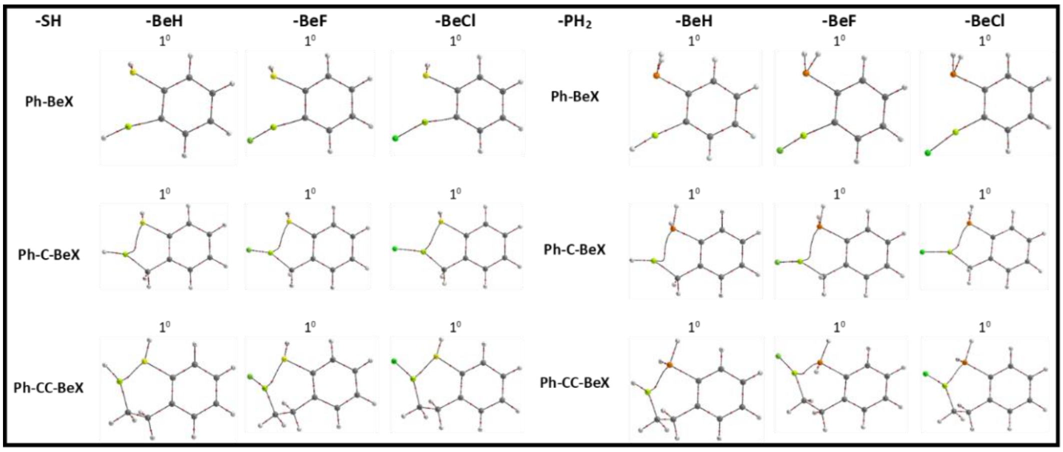

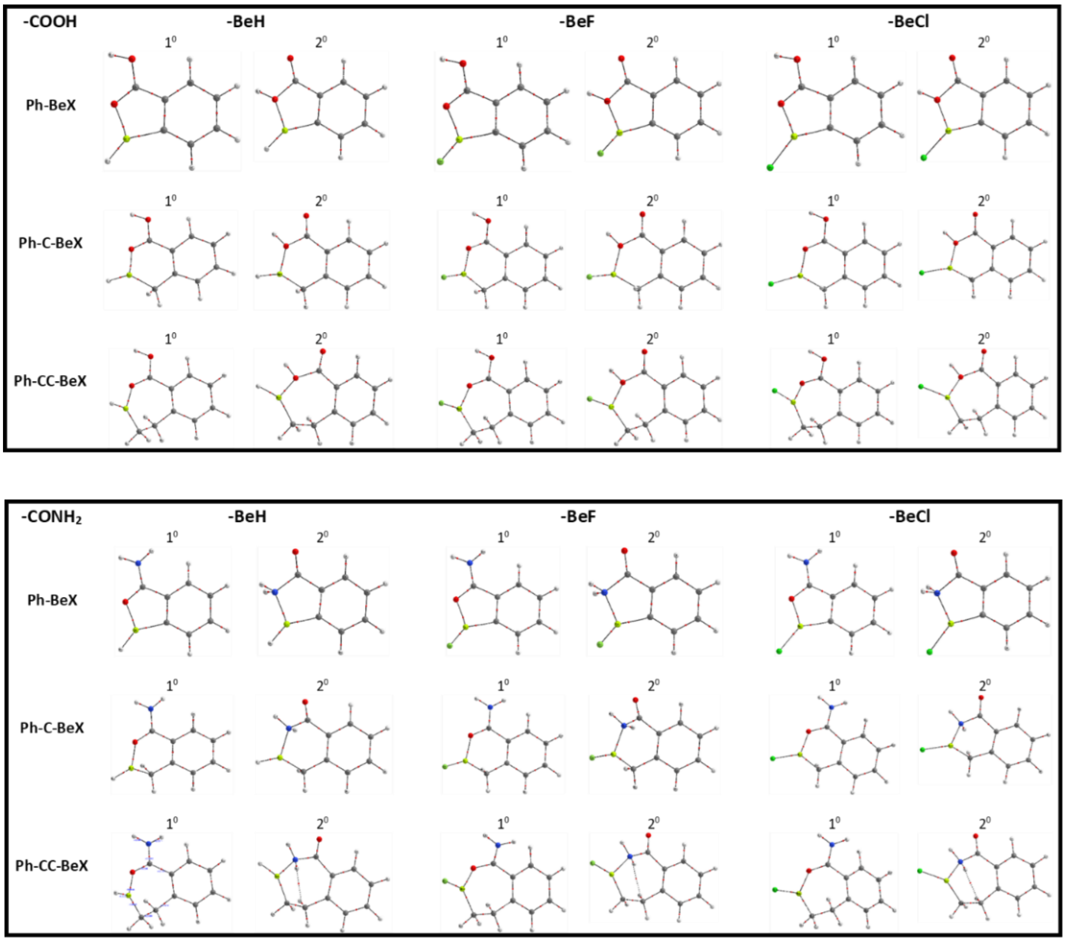

Figure S1. Molecular graphs for the beryllium-bonded complexes at the M06-2X/6-311+G(d,p) level of theory. Values for the corresponding topological parameters are detailed in Table S1.

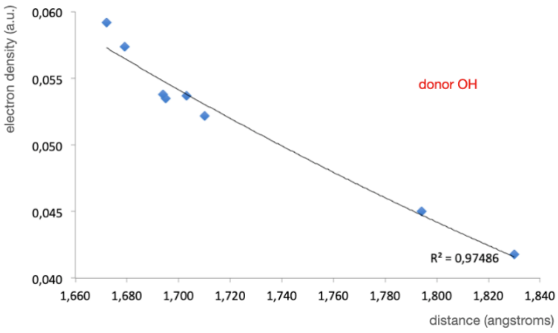

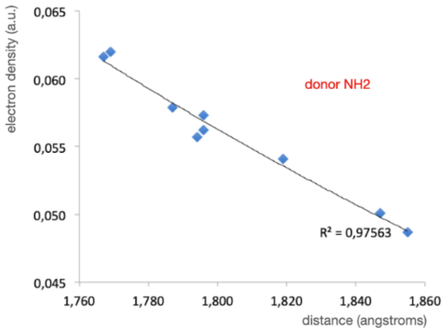

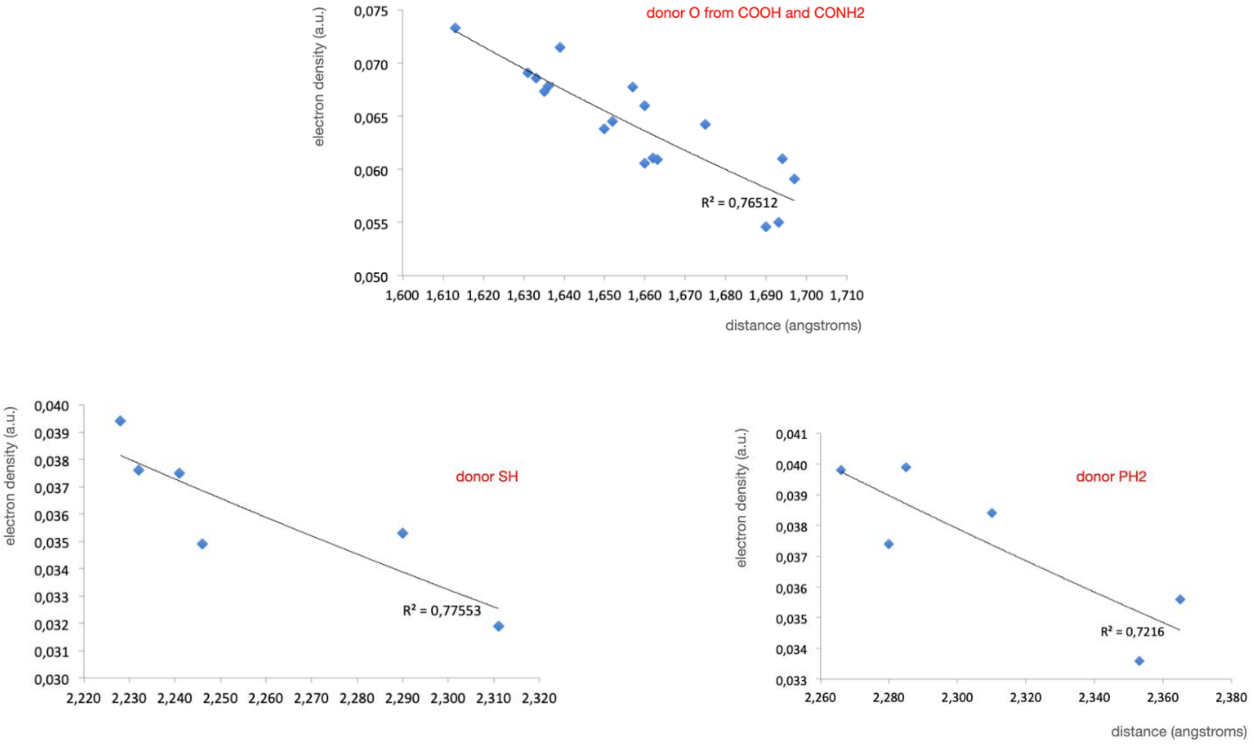

Figure S2. Exponential correlations between bond distances and electron energy densities for different donor groups.

Table S5. Cartesian coordinates for the Y:$(CH_2)_n$BeX 1,2-benzene derivatives (n = 0, 1, 2; X = H, F, Cl; Y = OH, NH_2, SH, PH_2, COOH, $CONH_2$) optimized at the M06-2X/6-31+G(d,p) level of theory.

ZERO CARBON

0_OH_BeCl_1

0,1

C,-1.0306857512,-0.6456410183,0.076230954

C,0.3469525338,-0.8057621443,-0.0962942787

C,1.1787002093,0.3148626512,-0.1812325988

C,0.5389367852,1.5403726068,-0.0944050379

C,-0.8134510715,1.7766691628,0.0698241628

C,-1.6034620698,0.6278286637,0.162511225

H,-1.6716963772,-1.5189609305,0.146329597

H,0.7621552227,-1.8079442279,-0.1551638616

H,-1.239041212,2.7740982632,0.1141767993

H,-2.6760317741,0.7288712301,0.294206655

Be,2.6677743122,1.1559381877,-0.265368409

O,1.5426270071,2.5527903137,-0.2636654818

H,1.5278649856,3.252462508,0.4040368435

Cl,4.4904642,1.406620734,-0.1880215691

0_OH_BeH_2

0,1
C,-0.8835058748,-0.5986492956,0.0000033315
C,0.5065775763,-0.5452127745,0.0004485955
C,1.2231626615,0.6734998299,-0.0000105427
C,0.4510702191,1.8467282455,-0.0009463523
C,-0.9448886509,1.8188127671,-0.0014017136
C,-1.6049712364,0.5964375182,-0.0009245386
H,-1.3981853621,-1.5535261521,0.0003739968
H,1.0534860677,-1.4877477752,0.001172475
H,-1.4878903516,2.7579411164,-0.0021245148
H,-2.6906346675,0.5770327166,-0.0012820582
Be,2.8793834319,0.468122822,0.0006409099
H,4.188333671,0.248661887,0.002240024
O,1.0105450564,3.092689331,-0.0014729367
H,1.9717870395,3.0279986434,-0.0011024971

0_OH_BeF_1
0,1
C,-0.9984248986,-0.6439050632,0.0709888127
C,0.3816822375,-0.7996396309,-0.0873302935
C,1.2131138622,0.3215571013,-0.1634608548
C,0.5727151372,1.5480420408,-0.0833802224
C,-0.7833648298,1.7773195202,0.065831385
C,-1.573815309,0.6282198367,0.15072738
H,-1.6378679505,-1.5188732066,0.1347949725
H,0.7991034061,-1.8012503319,-0.1417596183
H,-1.2117848072,2.7739137669,0.1050502204
H,-2.6478401816,0.7285050877,0.270781561
Be,2.7181216419,1.1450776168,-0.2509507962
O,1.5657713211,2.5662595605,-0.2389462288
H,1.5208255258,3.2701827364,0.42170829
F,4.1028702449,1.3567961354,-0.2308912576

0_OH_BeF_2
0,1
C,-0.8864368376,-0.6048941148,0.0000067272
C,0.5036403382,-0.5718864987,0.0005700126
C,1.2326202027,0.6378709056,0.0001276691
C,0.4800860988,1.8232403214,-0.0009228204
C,-0.9167214193,1.8121884651,-0.0014584634
C,-1.5927295379,0.5994505617,-0.0009911182
H,-1.4132840858,-1.5530264933,0.0003635178

H,1.0386038786,-1.5197984499,0.0013772081
H,-1.4465502582,2.7587142769,-0.002233512
H,-2.6784704735,0.5940901139,-0.0014284897
Be,2.8973393682,0.4970020781,0.000955339
O,1.0509424326,3.0637809806,-0.0013391727
H,2.0123815647,3.0005877563,-0.0008783512
F,4.2728482586,0.3854893472,0.0016270639

0_OH_BeCl_1
0,1
C,-1.0306857512,-0.6456410183,0.076230954
C,0.3469525338,-0.8057621443,-0.0962942787
C,1.1787002093,0.3148626512,-0.1812325988
C,0.5389367852,1.5403726068,-0.0944050379
C,-0.8134510715,1.7766691628,0.0698241628
C,-1.6034620698,0.6278286637,0.162511225
H,-1.6716963772,-1.5189609305,0.146329597
H,0.7621552227,-1.8079442279,-0.1551638616
H,-1.239041212,2.7740982632,0.1141767993
H,-2.6760317741,0.7288712301,0.294206655
Be,2.6677743122,1.1559381877,-0.265368409
O,1.5426270071,2.5527903137,-0.2636654818
H,1.5278649856,3.252462508,0.4040368435
Cl,4.4904642,1.406620734,-0.1880215691

0_OH_BeCl_2
0,1
C,-0.8784580825,-0.6055322078,0.0000104846
C,0.5111652807,-0.5647365078,0.0005836469
C,1.2341353928,0.6496827598,0.0001754547
C,0.4742585662,1.8311770523,-0.0008452945
C,-0.9224323366,1.8122395368,-0.0014396974
C,-1.5912180308,0.5953918247,-0.0010075999
H,-1.4003484816,-1.5563892657,0.0003455914
H,1.0522318879,-1.5095275341,0.0013670432
H,-1.4579957016,2.755543748,-0.0022342326
H,-2.6769236682,0.5838637476,-0.0014688939
Be,2.8922113733,0.5034425309,0.0009474327
O,1.0396638072,3.0735406633,-0.0013706157
H,2.0015041066,3.013366701,-0.0009365839
Cl,4.6864753468,0.3407760309,0.0018852545

0_NH2_BeH_1
0,1
C,-1.0357106028,-0.6456571895,-0.0002403427
C,0.3560900084,-0.7331470852,0.0001337846
C,1.1406366742,0.4291961947,0.0000431416
C,0.4448507508,1.6365906518,-0.0003758425
C,-0.93947074,1.7782797395,-0.0009444493
C,-1.6808745701,0.5985082442,-0.0009953485
H,-1.6359876127,-1.551147167,-0.0001812751
H,0.8234951716,-1.7148004184,0.0004362288
H,-1.424094602,2.7513850075,-0.0015359784
H,-2.765692133,0.6427935815,-0.0015396265
Be,2.6311298153,1.3063969357,-0.0000568916
N,1.4362505421,2.7252746092,-0.000638574
H,1.3900638153,3.3231604012,-0.8232695758
H,1.3894977491,3.3233698603,0.8218262656
H,3.972314174,1.4246659045,-0.000101656

0_NH2_BeH_2
0,1
C,-0.9004601491,-0.607682788,0.0445536691
C,0.4918226755,-0.6120655484,-0.0055225626
C,1.258380312,0.5692070115,-0.0508933995
C,0.5508105228,1.793059606,-0.0217204355
C,-0.8499996429,1.8094177308,0.01975352
C,-1.5653226231,0.6178619868,0.0501812587
H,-1.4563755023,-1.5386938496,0.0743971057
H,0.9986222625,-1.5762414015,-0.0183749572
H,-1.3773968319,2.7602338389,0.0313010906
H,-2.6504246335,0.6495776907,0.0849763479
Be,2.9089608179,0.4035302169,-0.2376033035
N,1.2447204783,3.00635358,-0.095538129
H,0.7071058612,3.8260437166,0.1503372806
H,2.1545306883,3.016516879,0.3443664973
H,4.220159974,0.2343250103,-0.3758642826

0_NH2_BeF_1
0,1
C,-1.0184108435,-0.6499601027,-0.0000083576
C,0.3734709384,-0.7377069712,0.0008753757
C,1.1567328941,0.424571013,0.0005300025
C,0.4639330792,1.6344345412,-0.0008212184

C,-0.9209299347,1.7735398873,-0.001724225
C,-1.6628694693,0.5941906862,-0.0012811697
H,-1.6184681129,-1.5553703749,0.000303282
H,0.8416365635,-1.718528052,0.0018979698
H,-1.405930659,2.7462645379,-0.0027676609
H,-2.7475004104,0.6400504225,-0.0019588039
Be,2.6361868915,1.3124667462,0.00071089
N,1.4505514981,2.728833831,-0.0012141104
H,1.3971304144,3.3261143364,-0.824126787
H,1.3967021806,3.327113611,0.8209408915
F,4.0402633599,1.4488755281,0.0013652114

0_NH2_BeF_2
0,1
C,-0.9017856692,-0.6080223053,0.0244399039
C,0.4902074124,-0.6301612821,-0.02377039
C,1.2647477871,0.5438372964,-0.054470553
C,0.5760255904,1.7764064728,-0.0082474456
C,-0.824389802,1.80806991,0.0302472462
C,-1.553137361,0.6244279845,0.0425624465
H,-1.4685286805,-1.5327490794,0.041472525
H,0.9865538154,-1.5984309818,-0.0491663624
H,-1.3404251028,2.7647484249,0.0552857477
H,-2.6378218486,0.667742684,0.0747229996
Be,2.919744781,0.4312710838,-0.2547342937
N,1.2906355679,2.9804746063,-0.0647066291
H,0.7503304608,3.8073767063,0.1502543615
H,2.1724174378,2.9858260593,0.4304384821
F,4.2905597714,0.33064647,-0.3998169087

0_NH2_BeCl_1
0,1
C,-1.0268990645,-0.6460735355,-0.0010660636
C,0.3642467794,-0.7407945534,-0.0004256185
C,1.1497594143,0.4197620586,0.0001565235
C,0.4609780715,1.6314981395,-0.0001659678
C,-0.9225479522,1.7787838884,-0.00082516
C,-1.6669373693,0.6009224813,-0.0012196045
H,-1.6312236526,-1.5484778263,-0.0014269177
H,0.8294609296,-1.7228176278,-0.0002266171
H,-1.4041127599,2.7530257844,-0.0010570893
H,-2.7513586695,0.6496878645,-0.0017354386

Be,2.616776418,1.3094045936,0.0002980386
Cl,4.4661090838,1.4684018925,-0.0000016415
N,1.461907437,2.7150313436,0.0000059175
H,1.4228570362,3.3137022983,-0.8230003731
H,1.4234826185,3.3128626683,0.8236476821

0_NH2_BeCl_2
0,1
C,-0.8914078684,-0.6140858098,-0.0377072085
C,0.5004943313,-0.6270445951,0.0015939484
C,1.2683283333,0.5524403685,0.0403913745
C,0.5701157904,1.7813055127,0.0124689819
C,-0.830899579,1.8032524357,-0.0165806207
C,-1.5509121764,0.6146261232,-0.0382565961
H,-1.4522394805,-1.5422219456,-0.0617944826
H,1.0044694735,-1.5918484722,0.0128893923
H,-1.3540453851,2.7562960399,-0.0273942557
H,-2.6360662897,0.65069623,-0.0634503206
Be,2.9183401226,0.4417196915,0.2279090133
N,1.2749791755,2.9889871289,0.0776596193
H,2.1620399606,3.004963293,-0.4074686196
H,0.7309440766,3.8152436465,-0.1294758744
Cl,4.7109925155,0.3171633527,0.4039626488

0_SH_BeH_1
0,1
C,-0.9027991591,-0.6031447534,0.0964342756
C,0.4857562933,-0.6517661168,-0.0115335769
C,1.2501679836,0.5206410106,-0.1372572313
C,0.539959495,1.7299603791,-0.1484997881
C,-0.8487066083,1.8096953144,-0.05930717
C,-1.5691451144,0.6244782149,0.072606439
H,-1.4731642906,-1.5218904953,0.1949162422
H,0.9803994778,-1.61966956,0.0043113365
H,-1.3533507684,2.770320465,-0.0955244077
H,-2.6515839337,0.6575156961,0.149928701
Be,2.910862591,0.835949043,-0.2721637622
H,4.2401260945,0.8315139345,-0.3419297501
S,1.5625710888,3.2042258193,-0.3685343406
H,1.6028218506,3.5886630487,0.9192880326

0_SH_BeH_2

0,1
C,-0.9334194529,-0.6518282834,-0.0000575747
C,0.4584078036,-0.5988289362,0.0004867066
C,1.1742206127,0.6157995881,0.0001403876
C,0.4057821905,1.7971308764,-0.0008166624
C,-0.9916500695,1.7620819373,-0.0013792235
C,-1.6560010487,0.5392715184,-0.0009969325
H,-1.4475966485,-1.6073342845,0.0002405134
H,1.00629161,-1.5400219744,0.0012177662
H,-1.5605717136,2.6877838975,-0.0021140658
H,-2.7415902285,0.5214727781,-0.0014407059
Be,2.8464387566,0.5159433099,0.0008797974
H,4.1696983486,0.4061113636,0.001501653
S,1.1285867217,3.4284224898,-0.0013995414
H,2.4156731179,3.0467857195,-0.0006491179

0_SH_BeF_1
0,1
C,-0.9707059473,-0.6169000804,0.0762465444
C,0.4230330382,-0.6113443507,0.0920303586
C,1.1429034611,0.5862809023,-0.0377363882
C,0.3908894562,1.7614099059,-0.1797321287
C,-1.0020916193,1.7847270161,-0.2155528015
C,-1.6814204049,0.575977016,-0.0779682222
H,-1.5108298214,-1.553278296,0.1786488577
H,0.9534255009,-1.5527312942,0.2070387387
H,-1.5406643759,2.7175464631,-0.3504992669
H,-2.766801355,0.5632452943,-0.0975193556
Be,2.7793801596,1.0279584239,-0.0650303558
S,1.3850524165,3.2540095214,-0.396809135
H,1.3409346361,3.6906797897,0.8745370635
F,4.1671358551,1.0730686885,-0.015727909

0_SH_BeF_2
0,1
C,-0.9269566346,-0.6552390106,0.0000181936
C,0.4647523667,-0.6145982376,0.0005821448
C,1.1854077435,0.5956095594,0.0001722214
C,0.4307287996,1.7850650949,-0.0008512649
C,-0.9676667551,1.758623714,-0.0014248614
C,-1.6406893021,0.5413493982,-0.0009886795
H,-1.4480590871,-1.6068830037,0.000359954

H,1.0061358319,-1.558522923,0.0013691287
H,-1.5296337232,2.6884200127,-0.0022144069
H,-2.7262974138,0.5315346656,-0.0014411751
Be,2.859225694,0.5328182423,0.0009345467
S,1.1613811215,3.4121060553,-0.0015006533
H,2.4474841802,3.0275009691,-0.0007982815
F,4.2384561787,0.4850254634,0.0015601334

0_SH_BeCl_1
0,1
C,-0.9791207212,-0.6189553898,-0.0706164807
C,0.4146817878,-0.6240147303,-0.0762164204
C,1.1374344999,0.5718368001,0.0507005919
C,0.3843479361,1.7464533033,0.1766382153
C,-1.007407217,1.7875861463,0.202859061
C,-1.6874360392,0.5787670217,0.0688025096
H,-1.5248548669,-1.5524222059,-0.1702757329
H,0.9415612578,-1.5685073532,-0.1803134193
H,-1.5423608312,2.7241275575,0.3257132489
H,-2.7729293825,0.5683609532,0.0793494753
Be,2.7425308808,1.1184546552,0.0878535633
Cl,4.5667863709,1.1163807906,-0.0103036694
S,1.4288054037,3.2085073999,0.3905911391
H,1.4182039011,3.6441036617,-0.8826195917

0_SH_BeCl_2
0,1
C,-0.9205867556,-0.6557247986,0.0000242313
C,0.4707080307,-0.6085469818,0.0005951797
C,1.1869111542,0.60536604,0.0001777498
C,0.4260341252,1.7917215729,-0.0008587737
C,-0.9720930068,1.7586282295,-0.0014410949
C,-1.6393499137,0.5380447901,-0.0009989693
H,-1.4377469115,-1.6094972449,0.0003717426
H,1.017033465,-1.549929251,0.0013932788
H,-1.5384499489,2.6857614716,-0.0022388976
H,-2.72492982,0.523302355,-0.0014576728
Be,2.8546386235,0.5333692639,0.0009376866
S,1.1498933532,3.4215664754,-0.001521605
H,2.4378516849,3.0425120204,-0.0007353104
Cl,4.6543549199,0.4462670577,0.0017624539

0_PH2_BeH_1
0,1
C, -2.746696, 0.259549, 0.073260
C, -1.355980, 0.314917, 0.058680
C, -0.663626, 1.538800, -0.033218
C, -1.442111, 2.713668, -0.110338
C, -2.840362, 2.665349, -0.096199
C, -3.490871, 1.438587, -0.004429
H, -3.254140, -0.697975, 0.144874
H, -0.798644, -0.617562, 0.120349
H, -3.421088, 3.582538, -0.156620
H, -4.576116, 1.399972, 0.006629
P, -0.477498, 4.281547, -0.228642
H, -1.151072, 4.900633, -1.309780
H, -1.135289, 5.047449, 0.764604
Be, 1.021474, 1.680656, -0.056213
H, 2.348924, 1.619516, -0.062012

0_PH2_BeH_2
0,1
C, -2.761566, 0.262838, 0.069968
C, -2.401989, 0.985205, 1.210107
C, -1.724376, 2.213293, 1.131864
C, -1.413746, 2.698648, -0.160261
C, -1.768350, 1.978157, -1.302344
C, -2.443804, 0.760992, -1.188608
H, -3.285539, -0.683360, 0.166931
H, -2.661170, 0.572830, 2.183648
H, -1.516711, 2.364375, -2.287006
H, -2.716360, 0.207740, -2.082291
P, -0.526162, 4.304513, -0.439329
H, -1.367040, 5.126358, 0.358494
H, 0.445769, 4.162617, 0.586564
Be, -1.287957, 3.094540, 2.497355
H, -0.964071, 3.756848, 3.602684

0_PH2_BeF_1
0,1
C, -2.726259, 0.258206, 0.073260
C, -1.335869, 0.316802, 0.058428
C, -0.652746, 1.543936, -0.033588
C, -1.430308, 2.718373, -0.110862

C, -2.828529, 2.663996, -0.096296
C, -3.474265, 1.434809, -0.004333
H, -3.231152, -0.700682, 0.145014
H, -0.775184, -0.612967, 0.119962
H, -3.412346, 3.579014, -0.156620
H, -4.559392, 1.392385, 0.006951
P, -0.464295, 4.284194, -0.229167
H, -1.135440, 4.903831, -1.309695
H, -1.118867, 5.050216, 0.764160
Be, 1.029294, 1.703235, -0.057826
F, 2.410320, 1.624926, -0.062890

0_PH2_BeF_2
0,1
C,-2.741155,0.261515,0.089678
C,-2.388135,0.990063,1.227473
C,-1.729003,2.225768,1.139088
C,-1.429314, 2.717258, -0.152580
C, -1.781747, 1.989844, -1.291247
C, -2.437305, 0.762563, -1.171270
H, -3.250774, -0.692145, 0.190017
H, -2.636935, 0.576587, 2.202484
H, -1.545012, 2.380009, -2.277787
H, -2.707274, 0.204242, -2.062579
P, -0.561910, 4.334301, -0.430489
H, -1.341632, 5.116744, 0.462585
H, 0.481067, 4.148177, 0.515726
Be, -1.292646, 3.123307, 2.492012
F, -0.954533, 3.824123, 3.633603

0_PH2_BeCl_1
0,1
C,-2.717393,0.258167,0.073081
C,-1.327353,0.324407,0.057794
C,-0.651028,1.556090,-0.034537
C,-1.436401,2.725828,-0.111229
C,-2.833938,2.663837,-0.096179
C,-3.472028,1.430521,-0.004079
H,-3.216990,-0.703350,0.144912
H,-0.760174,-0.601605,0.118966
H,-3.423516,3.575186,-0.156135
H,-4.556814,1.381355,0.007687

P,-0.476727,4.294945,-0.229507
H,-1.148499,4.912841,-1.311883
H,-1.132334,5.059579,0.765546
Be,1.025378,1.722432,-0.059205
Cl,2.829935,1.607818,-0.065249

0_PH2_BeCl_2
0,1
C,-2.740796,0.262483,0.092380
C,-2.386703,0.992795,1.228551
C,-1.727017,2.229282,1.137894
C,-1.428466,2.718467,-0.155240
C,-1.782117,1.988967,-1.291917
C,-2.437797,0.761820,-1.169268
H,-3.250394,-0.690864,0.194475
H,-2.634159,0.582012,2.205277
H,-1.546519,2.376857,-2.279746
H,-2.708683,0.201956,-2.059308
P,-0.561314,4.334511,-0.440020
H,-1.339575,5.120452,0.451454
H,0.482922,4.152086,0.505717
Be,-1.292121,3.123692,2.487241
Cl,-0.851165,4.037381,3.979813

0_COOH_BeH_1
0,1
C,-0.9327336133,-0.6083930847,-0.0001201564
C,0.4639920034,-0.5764756638,0.0005282268
C,1.1606833765,0.6371237092,0.0002103855
C,0.354516767,1.7927983857,-0.000786911
C,-1.0438292215,1.8014370549,-0.0014627704
C,-1.6883470438,0.5713260101,-0.0011150816
H,-1.4487103224,-1.564916029,0.0001594851
H,1.0100269755,-1.5163898822,0.0013181293
H,-1.6026232403,2.7329160423,-0.0022477646
H,-2.7723005327,0.5226435067,-0.0016118993
C,1.186863543,2.9910639334,-0.0010502615
O,2.4336059642,2.8785637879,-0.0004580762
O,0.6266485664,4.180488088,-0.0024320069
H,1.3237177667,4.8567432118,-0.0026187428
Be,2.822682251,1.2265697824,0.0007500159
H,4.10876543,0.8302017071,0.0016352382

0_COOH_BeH_2
0,1
C,-0.9203699243,-0.5837924503,0.0001835919
C,0.4756138814,-0.5774813285,0.0010963047
C,1.1995674469,0.6219085391,0.0005469523
C,0.4234061625,1.7971404285,-0.0009475589
C,-0.9739927769,1.8267808872,-0.0018966351
C,-1.6476699548,0.6124085068,-0.0013146044
H,-1.4549911739,-1.5296185783,0.000633992
H,1.0036069078,-1.5273884842,0.0022509536
H,-1.504484352,2.7745014618,-0.0030424788
H,-2.7324857688,0.5898632741,-0.0020175337
C,1.1851646827,3.0528504161,-0.0014820573
O,0.860154916,4.2010844222,-0.0028595877
O,2.5702385043,2.7197661474,-0.0004578255
H,3.1392335324,3.5031448233,-0.0007713937
Be,2.8806191874,1.0588627251,0.0011831549
H,4.1679781293,0.6735899997,0.0022706455

0_COOH_BeF_1
0,1
C,-0.9155431357,-0.6114546108,-0.0000098172
C,0.4809753297,-0.5802435833,0.0005758829
C,1.1759025535,0.6338615204,0.0001601578
C,0.3718865766,1.7912838651,-0.0008821088
C,-1.0267119294,1.7985990052,-0.0014875154
C,-1.6709964985,0.5685416414,-0.0010354626
H,-1.4318315829,-1.5676043908,0.0003355586
H,1.0266531049,-1.519832664,0.0013730462
H,-1.5848385205,2.7301919833,-0.0022883693
H,-2.7548657349,0.5197335996,-0.0014765485
C,1.200957674,2.9936265353,-0.0012650341
O,2.4477207739,2.888465977,-0.0006302995
O,0.633356421,4.1792546301,-0.0021448428
H,1.3253380275,4.8609537113,-0.0022296751
Be,2.8261117641,1.2370911844,0.0005237132
F,4.1788437967,0.8332525258,0.0013405544

0_COOH_BeF_2
0,1
C,-0.9049984794,-0.5878904051,0.00063403

C,0.4907599728,-0.5832623666,0.0014691991
C,1.2143151918,0.6156701379,0.0005594821
C,0.4418437004,1.7940534511,-0.0012058869
C,-0.955714242,1.8229116872,-0.0020832976
C,-1.6304811969,0.6091531104,-0.001140033
H,-1.4404935282,-1.5331519275,0.0013687024
H,1.0177819071,-1.5334480782,0.0028471669
H,-1.4856191731,2.770837944,-0.0034445027
H,-2.7153346726,0.5881664673,-0.0017626652
C,1.1995246339,3.0542940089,-0.0021279743
O,0.8603353759,4.1986960782,-0.0034801097
O,2.5847261344,2.7376896913,-0.0004826559
H,3.1450741705,3.527801245,-0.0008719592
Be,2.8867253624,1.0604184793,0.0010473355
F,4.243144193,0.6717016469,0.0022105085

0_COOH_BeCl_1
0,1
C,-0.9023681707,-0.6081112478,0.0000072582
C,0.4939500457,-0.5669657842,0.0006088801
C,1.1767266273,0.6531650028,0.0001855657
C,0.3644118377,1.8051695624,-0.0008201864
C,-1.034089622,1.8020516788,-0.0014776912
C,-1.667110555,0.5661184858,-0.0010426472
H,-1.4116428494,-1.5679195651,0.0003373881
H,1.0492718114,-1.5008612325,0.0013906114
H,-1.6003399731,2.7287024451,-0.0022672238
H,-2.750500815,0.5079952365,-0.0015071712
C,1.190099131,3.0074875117,-0.0010831322
O,2.4401444879,2.8987605813,-0.0004606538
O,0.629202483,4.1936851134,-0.0023041865
H,1.3227844508,4.8741338963,-0.00243585
Be,2.8127260616,1.2660792234,0.0004352426
Cl,4.5796935991,0.6962598518,0.0015294162

0_COOH_BeCl_2
0,1
C,-0.8916583202,-0.5857792549,-0.0000668222
C,0.5039332397,-0.5699626533,0.0004970818
C,1.2146298954,0.6359728128,0.0001081037
C,0.4328438047,1.8078155772,-0.0008437758
C,-0.9645094615,1.8254862524,-0.0014126853

C,-1.6272739409,0.605010622,-0.0010115348
H,-1.4196409072,-1.5352585946,0.0002180681
H,1.0414330717,-1.5144455838,0.0012228742
H,-1.5039722458,2.7682182918,-0.0021055223
H,-2.7119103102,0.5739454751,-0.0014164824
C,1.1854183878,3.0697867085,-0.0011865097
O,0.8549592765,4.2148070848,-0.0017880385
O,2.5783918851,2.746624701,-0.0006204458
H,3.1502961941,3.5291250321,-0.0004391571
Be,2.8738769971,1.097145156,0.0004915035
Cl,4.6447717137,0.5451793728,0.0021270626

0_CONH2_BeH_1
0,1
C,-0.9289876759,-0.6490111095,-0.0000207943
C,0.4655550491,-0.5922674531,0.0005457996
C,1.1468944739,0.6315639742,0.0001098925
C,0.3347349961,1.7810782047,-0.0009340679
C,-1.0636751638,1.7586167833,-0.0015168571
C,-1.6965885504,0.5217172563,-0.0010520258
H,-1.4327771969,-1.6118156118,0.0003358266
H,1.0274896804,-1.5228855985,0.0013421177
H,-1.6651965427,2.665862141,-0.0023174238
H,-2.7801897127,0.4637858282,-0.0014850945
Be,2.8054196984,1.2335235599,0.0004739773
C,1.1694993784,2.9993983134,-0.0013137229
O,2.4259923731,2.8493159644,-0.0006792273
N,0.6582455242,4.2285770605,-0.0022572636
H,1.2878454659,5.018203257,-0.0024622665
H,-0.3360065157,4.3877867746,-0.0027828325
H,4.0841148686,0.8030815457,0.0012770425

0_CONH2_BeH_2
0,1
C,-0.9360312699,-0.6034750333,0.0578810763
C,0.4569395462,-0.6156409239,0.0171934902
C,1.1981600422,0.5760208533,-0.0306584695
C,0.4405141918,1.7646962071,-0.0374875366
C,-0.9599960795,1.8075461119,-0.0111566377
C,-1.6479538856,0.6049347232,0.0430126579
H,-1.4838827824,-1.5410246715,0.0979443914
H,0.9737838453,-1.5718543301,0.0196934545

H,-1.4784244795,2.7618690174,-0.0315058359
H,-2.7328310139,0.5960262495,0.0710028078
Be,2.8865920322,0.9715040424,-0.1346860541
C,1.2208169913,3.0088859292,-0.1136884734
O,0.8505137103,4.1337689797,-0.3204695518
N,2.6745770444,2.7195735175,0.0703285336
H,3.2159141096,3.3811073781,-0.4867914766
H,2.9166481665,2.9186097718,1.0448679586
H,4.1286517212,0.4587921676,-0.2612385149

0_CONH2_BeF_1
0,1
C,-0.913481256,-0.6509398544,0.0000315945
C,0.4808267971,-0.5955143672,0.0006332481
C,1.1608810999,0.6285283645,0.0001817188
C,0.3517945626,1.7800120706,-0.0008457404
C,-1.0469030539,1.7570614715,-0.0015063863
C,-1.6800686101,0.5205336617,-0.0010445357
H,-1.4177680015,-1.613372591,0.0003807303
H,1.0424130509,-1.5260124323,0.0014401427
H,-1.6485560013,2.6639830235,-0.0023371924
H,-2.7636508087,0.4634064338,-0.0015150101
Be,2.8090046476,1.2439843702,0.0004873222
C,1.1839973111,3.0023324702,-0.0010966523
O,2.4406440614,2.8593271738,-0.00068081
N,0.6661544476,4.2284486225,-0.0023440569
H,1.2915856081,5.0215039381,-0.0027062011
H,-0.3288730016,4.3825741151,-0.0030189232
F,4.1543692466,0.8006947895,0.0013652619

0_CONH2_BeF_2
0,1
C,-0.9226394776,-0.6134232239,0.0065054137
C,0.4700615808,-0.6258055199,-0.0366300426
C,1.2111222469,0.5660916573,-0.0476345141
C,0.4570561257,1.7570438772,-0.0144224447
C,-0.9439017674,1.7981541061,0.0191070754
C,-1.6330609615,0.595428228,0.0340459626
H,-1.4711916538,-1.5513415833,0.0160763806
H,0.9856278418,-1.5820978668,-0.0647643496
H,-1.4616334101,2.7528472859,0.0342071456
H,-2.717877269,0.5870037907,0.0649412926

Be,2.8921550732,0.9812071458,-0.1264198455
F,4.2054794214,0.4590262494,-0.2488570254
C,1.228746305,3.0096299578,-0.0497373784
O,0.8379119152,4.13822099,-0.1855839953
N,2.692432891,2.7362215374,0.0816286151
H,3.2000538965,3.3834252784,-0.5229539815
H,2.9736490818,2.9797284499,1.0348949417

0_CONH2_BeCl_1
0,1
C,-0.8974260352,-0.6495078397,-0.0001463868
C,0.4963225157,-0.5830909813,0.0004354127
C,1.1626839047,0.6473563142,0.000063879
C,0.3446482911,1.7927860441,-0.0009255099
C,-1.0534357227,1.758235293,-0.0015269981
C,-1.6740412214,0.5153999078,-0.0011264864
H,-1.3942460964,-1.6158309589,0.000164063
H,1.0685903768,-1.5071688815,0.0011955931
H,-1.6628929446,2.6599339731,-0.0022889771
H,-2.7570627785,0.4479589331,-0.0015710821
Be,2.7934914964,1.2742254811,0.0005398046
C,1.1714672701,3.0163557688,-0.0012118196
O,2.4319625353,2.8731362264,-0.0005508101
N,0.6581816268,4.2420442772,-0.0021248314
H,1.2861027752,5.0333594871,-0.0022764487
H,-0.3365200276,4.3992256953,-0.0027192941
Cl,4.5545440642,0.6621623501,0.0017307818

0_CONH2_BeCl_2
0,1
C,-0.9079542168,-0.6145283236,0.0146428345
C,0.485304376,-0.6148463449,0.0024955533
C,1.212269166,0.5850165015,-0.0104375625
C,0.4482046741,1.7696938783,-0.0114155676
C,-0.9534101952,1.7979815252,-0.0035686088
C,-1.6299986032,0.5881428998,0.0115618021
H,-1.4481675812,-1.557102325,0.0254944266
H,1.0130088067,-1.5648589087,0.0021095321
H,-1.4809470147,2.7473173035,-0.0086130906
H,-2.7150253709,0.5690837968,0.020215951
Be,2.8798364276,1.0116934477,-0.0389662707
C,1.2112097634,3.0258565555,-0.0348216565

O,0.817804903,4.158358882,-0.1020427331
N,2.6886973265,2.7507475296,0.0263112375
H,3.1477911526,3.2905194732,-0.7103261265
H,3.0478130833,3.1439704198,0.8999981903
Cl,4.5975550726,0.284343879,-0.0879982813

ONE CARBON

1_OH_BeH_1
0,1
C,0,-4.1294394789,-0.3700954238,-0.3261635577
C,0,-2.7416670236,-0.4968068189,-0.2316272557
C,0,-1.9326153988,0.6056268395,0.0521142731
C,0,-2.5988240835,1.8167481661,0.2308263492
C,0,-3.967678453,1.9944932772,0.1482568185
C,0,-4.7414154494,0.8679018534,-0.137537234
H,0,-4.7367568907,-1.241765145,-0.5480234876
H,0,-2.2718773266,-1.4654507501,-0.3796905104
H,0,-4.4257840681,2.9681996245,0.2986154875
H,0,-5.8194452033,0.9643739354,-0.2112532492
O,0,-1.7055066354,2.8669461499,0.5111863347
H,0,-2.0980445006,3.7375928379,0.6411985401
C,0,-0.4285268554,0.6645840879,0.1926475936
H,0,0.0440859514,0.2971048435,-0.7278053945
H,0,-0.1041460382,-0.0237049507,0.9847125869
Be,0,-0.094135915,2.3415916292,0.5417430209
H,0,0.8055895991,3.3147067339,0.8031160445

1_OH_BeH_2
0,1
C,0,-4.1140874586,-0.3565747179,0.0087168697
C,0,-2.7210852696,-0.3700215476,0.0159364507
C,0,-1.9634409867,0.8057232162,0.0084985871
C,0,-2.6681691951,2.0216647749,-0.0067939216
C,0,-4.0620068267,2.0548853945,-0.0141733251
C,0,-4.782765339,0.865894635,-0.0064078015
H,0,-4.6680728688,-1.2891515664,0.0148444947
H,0,-2.2084609295,-1.3319079126,0.027771836
H,0,-4.5561573429,3.0207187635,-0.025958044
H,0,-5.8677976613,0.8964950155,-0.0122049782
O,0,-2.0219329878,3.2208773703,-0.0150800921

H,0,-1.0688562711,3.0719113896,-0.0088669531
C,0,-0.4481991822,0.7975817031,0.0162953347
H,0,-0.057071441,1.3324377006,-0.8659369743
H,0,-0.0660346387,1.349861789,0.8916968725
Be,0,0.2646329227,-0.7353919979,0.0352433925
H,0,0.8495201762,-1.9276931,0.050322752

1_OH_BeF_1
0,1
C,0,-4.1185117711,-0.3653430797,-0.3246724619
C,0,-2.7313737499,-0.4880042112,-0.2195306394
C,0,-1.9269352077,0.6164193572,0.0690086262
C,0,-2.5958399673,1.8281546007,0.2414290463
C,0,-3.9650959342,1.9999466431,0.147472434
C,0,-4.7341248727,0.8715662878,-0.1424200901
H,0,-4.7223310695,-1.2385897601,-0.5498287923
H,0,-2.257759494,-1.4555152154,-0.3628266647
H,0,-4.4265058123,2.9728614103,0.2929553408
H,0,-5.8117514044,0.9654277784,-0.2243717033
O,0,-1.7145173023,2.8845874274,0.527829264
H,0,-2.1152628517,3.7518221717,0.6581129197
C,0,-0.422755861,0.6643887983,0.2180386995
H,0,0.0518008179,0.3023858635,-0.7027185313
H,0,-0.1071710432,-0.0285555366,1.0081555602
Be,0,-0.0953257821,2.3369611561,0.5720556283
F,0,0.8404675554,3.3635685885,0.857469094

1_OH_BeF_2
0,1
C,0,-4.1297326922,-0.3606226983,0.1125183189
C,0,-2.7378584988,-0.3559441214,0.0591606112
C,0,-2.00089286,0.8259825246,-0.0635091382
C,0,-2.723361248,2.0293600528,-0.1323550733
C,0,-4.1164280985,2.0439123791,-0.0805002217
C,0,-4.8172406483,0.8494002581,0.04180328
H,0,-4.6676860745,-1.2975307472,0.2079888831
H,0,-2.2106586785,-1.3089255426,0.1153239808
H,0,-4.6257681176,3.0001998134,-0.1376416197
H,0,-5.9017792648,0.8654253563,0.0818042471
O,0,-2.098148615,3.2338296962,-0.252543718
H,0,-1.143021031,3.1029782197,-0.2783525326
C,0,-0.4865147715,0.8435546221,-0.1225692986

H,0,-0.1415081828,1.3148270452,-1.0565291832
H,0,-0.0748432723,1.455537045,0.6956583699
Be,0,0.2194118441,-0.6890189706,-0.025674868
F,0,0.8050708595,-1.9362999223,0.0529457724

1_OH_BeCl_1
0,1
C,0,-4.4282182707,0.0521494495,-0.9394700853
C,0,-3.0917570968,-0.2668804417,-1.18882221
C,0,-2.0592455599,0.34721108,-0.4769645249
C,0,-2.4526758228,1.2814525172,0.4795973974
C,0,-3.7590670527,1.6321703371,0.7641085059
C,0,-4.7621751685,0.9956569845,0.0306210271
H,0,-5.2144561105,-0.4376816913,-1.5047791186
H,0,-2.8391919651,-1.0034315236,-1.9466498896
H,0,-3.9984907052,2.3702692316,1.5244113732
H,0,-5.8013484802,1.2410683107,0.2216779584
O,0,-1.3463114948,1.8467776025,1.144960568
H,0,-1.5347155463,2.5045244944,1.825490221
C,0,-0.5674521401,0.1309679669,-0.6081458621
H,0,-0.3278362984,-0.9253173078,-0.4327519331
H,0,-0.2501039873,0.3419515139,-1.6370844122
Be,0,0.099069767,1.2119105976,0.5719459031
Cl,0,1.6197034823,1.9075679585,1.4031887219

1_OH_BeCl_2
0,1
C,0,-4.1192929705,-0.3593588883,-0.0197372028
C,0,-2.7267275929,-0.3836468383,-0.0292751555
C,0,-1.961186793,0.7865888738,-0.0131676643
C,0,-2.6551795578,2.0084998922,0.0101564747
C,0,-4.0483899511,2.0517941364,0.0237845053
C,0,-4.7780655668,0.8684678642,0.0087733339
H,0,-4.6799021368,-1.2877341211,-0.0330019819
H,0,-2.2209794927,-1.349316737,-0.0517092978
H,0,-4.5357545852,3.020920237,0.0447877818
H,0,-5.8627309163,0.9072788752,0.0180051069
O,0,-2.0016525442,3.2038980029,0.0216752159
H,0,-1.0498880323,3.0539931834,-0.0183784705
C,0,-0.4455993981,0.7740145087,-0.0204106141
H,0,-0.0580171005,1.2652533483,-0.927660389
H,0,-0.053268264,1.358544735,0.8281962499

Be,0,0.2470512762,-0.7595451365,0.0819045127
Cl,0,1.0196323761,-2.382452166,0.2131962949

1_NH2_BeH_1
0,1
C,0,-4.1294864002,-0.3801246576,-0.3153998057
C,0,-2.7432348071,-0.4548989998,-0.2083318541
C,0,-1.9722531617,0.6840217812,0.0623877294
C,0,-2.6655107798,1.8892975917,0.2177786764
C,0,-4.0501762819,1.9895880775,0.1154089521
C,0,-4.7884024428,0.8409723143,-0.1542009477
H,0,-4.7036693729,-1.2775037801,-0.5258138079
H,0,-2.2390309169,-1.409268001,-0.3352461957
H,0,-4.544772573,2.9493782102,0.2441334374
H,0,-5.8687732258,0.8971667161,-0.2380348764
C,0,-0.4688096752,0.7410866727,0.2030743456
H,0,0.003119709,0.3600205437,-0.7131548083
H,0,-0.1453568733,0.0544472387,0.9976317614
Be,0,-0.1366397599,2.4210206299,0.5322313565
N,0,-1.8112800445,3.0440298724,0.4985406768
H,0,-1.9100436816,3.7709957774,-0.2104607005
H,0,-2.0484702582,3.4847707679,1.3875973289
H,0,0.8295963461,3.3422763848,0.7807884017

1_NH2_BeH_2
0,1
C,0,-3.9674406637,-0.3386121697,0.2120015784
C,0,-2.5850071845,-0.337327984,0.0650399935
C,0,-1.8606312845,0.8441767241,-0.1268717707
C,0,-2.5716478448,2.0646902123,-0.1471284839
C,0,-3.962862315,2.0610222844,-0.0041472946
C,0,-4.6557634564,0.8708099378,0.1733489178
H,0,-4.5003101059,-1.272715808,0.3525308334
H,0,-2.039524035,-1.2781374654,0.0859991266
H,0,-4.4977618108,3.006778174,-0.0351970033
H,0,-5.7350823453,0.8913735492,0.2851775507
C,0,-0.3541972925,0.8488629741,-0.2150597026
H,0,0.0119475183,1.5363550011,-0.9828692288
H,0,0.0106589497,-0.1514365582,-0.46978919
Be,0,-0.0466417997,1.2011473016,1.4057539285
H,0,0.0876792155,1.4751740749,2.697827209
N,0,-1.8918006021,3.2641640989,-0.3694289641

H,0,-2.3719897466,4.0911370914,-0.0459357327
H,0,-0.9101619068,3.2726310613,-0.1343948471

1_NH2_BeF_1
0,1
C,0,-4.1187484856,-0.3735895072,-0.3157441998
C,0,-2.7339173299,-0.4470275391,-0.1939615545
C,0,-1.9650544146,0.6922753453,0.0813426875
C,0,-2.6598497948,1.8994434489,0.2262219197
C,0,-4.0439377106,1.9967569853,0.1087209507
C,0,-4.7792859081,0.8477807813,-0.1650241668
H,0,-4.6902149572,-1.2719215402,-0.52944125
H,0,-2.2278983579,-1.4014766969,-0.3127225523
H,0,-4.5400401081,2.9567892119,0.2294301645
H,0,-5.8586884474,0.9037210507,-0.2601689798
C,0,-0.4621039117,0.7353211554,0.2351328768
H,0,0.0136321319,0.3513092772,-0.6768490657
H,0,-0.1529511207,0.0501463892,1.0354179838
Be,0,-0.1433693079,2.4156486426,0.5616918406
N,0,-1.8182011112,3.0615562117,0.5125977357
H,0,-1.915216005,3.7836680184,-0.2013559954
H,0,-2.0706502257,3.5023090847,1.3972398197
F,0,0.8664948944,3.3846022108,0.830242835

1_NH2_BeF_2
0,1
C,0,-4.1267819563,-0.3839054092,-0.0216078924
C,0,-2.7338051225,-0.3633213289,-0.0686305267
C,0,-1.9995887046,0.8239955994,-0.0385854618
C,0,-2.7138151714,2.0397251633,0.034436466
C,0,-4.1130864245,2.0246620027,0.0869679329
C,0,-4.8138902965,0.8241921656,0.0604819371
H,0,-4.6617234638,-1.3270260729,-0.0458537045
H,0,-2.2016306688,-1.3132818099,-0.1318801696
H,0,-4.6504724083,2.9676778601,0.1524047098
H,0,-5.8987461644,0.836642127,0.1007768828
C,0,-0.4861389785,0.8384699739,-0.0752158495
H,0,-0.128853402,1.3055953205,-1.0076292137
N,0,-2.0227683288,3.2497110524,0.1142350818
H,0,-1.1143810166,3.2635947211,-0.3272565704
H,0,-2.5698780451,4.0693492666,-0.1069437992
H,0,-0.1058152122,1.4804628086,0.734038042

Be,0,0.2243985439,-0.6862004351,0.0716126097
F,0,0.8190110505,-1.928447745,0.1827906557

1_NH2_BeCl_1
0,1
C,0,-4.1213443713,-0.3681534243,-0.3669156246
C,0,-2.7385936147,-0.4465908637,-0.2247951113
C,0,-1.9757392917,0.6866596071,0.0878540825
C,0,-2.6727095281,1.8893177669,0.247844004
C,0,-4.053910799,1.9932467217,0.1109661156
C,0,-4.7838803731,0.8500357382,-0.1999329639
H,0,-4.6904165579,-1.2605621684,-0.6095358328
H,0,-2.2314752086,-1.3986205069,-0.3565107588
H,0,-4.5514764822,2.95064074,0.2448558872
H,0,-5.8615422224,0.9081242588,-0.3112956754
C,0,-0.4748914397,0.7333150315,0.2682852074
H,0,0.0211167978,0.3737939071,-0.6427592208
H,0,-0.1730133929,0.0348533355,1.0596811467
Be,0,-0.1810935451,2.4003214201,0.6336541971
Cl,0,1.1561399657,3.654236341,1.0356759572
N,0,-1.8285614959,3.0424018928,0.5736782812
H,0,-1.9104565551,3.782773091,-0.1243984227
H,0,-2.091689376,3.4665714717,1.4641875111

1_NH2_BeCl_2
0,1
C,0,-3.9849973611,-0.3505530409,0.1919286599
C,0,-2.5970683856,-0.3302848187,0.0829466793
C,0,-1.8834163226,0.8609502794,-0.0810293158
C,0,-2.6069214989,2.070736868,-0.1270524929
C,0,-4.00282878,2.05033607,-0.0221153527
C,0,-4.6880385746,0.8514705551,0.1371368022
H,0,-4.5086367208,-1.2926466288,0.3143738038
H,0,-2.0422176788,-1.2654124097,0.1184036978
H,0,-4.5491986878,2.9891136467,-0.0716196298
H,0,-5.7704304215,0.8594320741,0.2175109794
C,0,-0.3746160846,0.8715062714,-0.1507449351
H,0,-0.0102203015,1.5447107096,-0.9357186925
H,0,-0.0105025904,-0.1300727636,-0.4078251874
Be,0,0.1530913846,1.2598752021,1.4036638891
N,0,-1.9409774837,3.2822167347,-0.3393337566
H,0,-2.4487637183,4.1069018112,-0.0525122381

H,0,-0.968485129,3.3204279959,-0.0706458613

1_SH_BeH_1
0,1
C,0,-4.1355599467,-0.3892298502,-0.1689551855
C,0,-2.7519861639,-0.4126734669,-0.0289446491
C,0,-1.9982614386,0.7689162379,0.0481711181
C,0,-2.7216676792,1.9703283255,-0.0126734116
C,0,-4.1046219041,2.0168956269,-0.1875428885
C,0,-4.8185382013,0.8256838236,-0.2545585922
H,0,-4.6868072922,-1.323320919,-0.2205675528
H,0,-2.2294775848,-1.3640170281,0.0232488304
H,0,-4.6139849502,2.9730518795,-0.260737673
H,0,-5.8966509407,0.8446892118,-0.374855562
C,0,-0.4990155069,0.794035153,0.1904683272
H,0,-0.056708226,0.6267931536,-0.8068866726
H,0,-0.1624705414,-0.055437863,0.7950729108
Be,0,0.0692944086,2.3459391629,0.7081491359
H,0,1.035516169,3.0935969978,1.2714629606
S,0,-1.7597109034,3.4883485712,0.0968012152
H,0,-2.1390122082,3.8843617235,1.326334069

1_SH_BeH_2
0,1
C,0,-4.1251731873,-0.3344412911,0.0223252795
C,0,-2.7326874625,-0.3595499708,0.0286420743
C,0,-1.9633954446,0.8078130247,-0.0233299304
C,0,-2.6530493114,2.0344830574,-0.0834822272
C,0,-4.0496984962,2.0703364879,-0.090309627
C,0,-4.7845265963,0.8896496777,-0.0375743333
H,0,-4.6867763675,-1.2618829213,0.0637906815
H,0,-2.2264821314,-1.3228736233,0.0757250282
H,0,-4.5683841071,3.0238185361,-0.136994099
H,0,-5.8690389122,0.9321194806,-0.043563527
C,0,-0.451940575,0.7674949902,-0.0158849177
H,0,-0.0734162657,1.2913061914,-0.9097138321
S,0,-1.6787770128,3.5209572218,-0.1497676166
H,0,-2.722424194,4.3624722045,-0.1931227647
H,0,-0.0797932575,1.3736503816,0.8271382207
Be,0,0.2916833999,-0.745822688,0.0587682108
H,0,0.9060982915,-1.9231386894,0.1171228498

1_SH_BeF_1
0,1
C,0,-4.1234866305,-0.3810879619,-0.1558494467
C,0,-2.7408971613,-0.4023628277,-0.0102764486
C,0,-1.9857670751,0.7792055348,0.0564531194
C,0,-2.7066032483,1.9823331774,-0.0219332508
C,0,-4.0899635254,2.0238083517,-0.1997491229
C,0,-4.8048236304,0.8332120914,-0.2568943203
H,0,-4.6749381621,-1.3153913699,-0.1996993063
H,0,-2.2187619153,-1.3530792683,0.0550121353
H,0,-4.5990650618,2.9789358487,-0.2869539007
H,0,-5.8823587569,0.8519792691,-0.3827448852
C,0,-0.4869592345,0.7874529211,0.20585517
H,0,-0.038873983,0.6325268442,-0.7890765631
H,0,-0.1632391536,-0.0693497574,0.8064576261
Be,0,0.063416,2.3240808054,0.7707419074
S,0,-1.7646224216,3.5135215225,0.071362741
H,0,-2.1936821485,3.9387151385,1.2746948295
F,0,1.0341572284,3.1034958204,1.4304274757

1_SH_BeF_2
0,1
C,0,-4.1424357395,-0.3162491951,0.0157207302
C,0,-3.5547265113,0.1288035172,-1.1657550311
C,0,-2.7398953191,1.2666891381,-1.2167085225
C,0,-2.5522164594,1.9763129249,-0.0131150259
C,0,-3.1421843563,1.5370817529,1.175007684
C,0,-3.9276204012,0.3885702951,1.1958691307
H,0,-4.760369646,-1.2083825421,0.0103395921
H,0,-3.7327618589,-0.4367377888,-2.0801961931
H,0,-2.9917126392,2.1116332819,2.0839299512
H,0,-4.3764861743,0.0571700281,2.1266743277
C,0,-2.1011882814,1.7111041898,-2.513269569
H,0,-2.6032676323,2.618217123,-2.8856240226
S,0,-1.5044362179,3.4175791953,0.0966127594
H,0,-1.914513784,4.0074225805,-1.0371532367
H,0,-1.0658693808,2.0236370795,-2.3175735946
Be,0,-2.1003228393,0.5529291922,-3.7431409813
F,0,-2.1115886393,-0.3818013226,-4.7595306284

1_SH_BeCl_1
0,1

C,0,-4.1243323752,-0.3871107917,-0.2134680898
C,0,-2.7452347552,-0.4037007857,-0.0361930594
C,0,-2.0015386735,0.7810310783,0.0770026848
C,0,-2.7307718704,1.9788760332,0.0111978201
C,0,-4.1096356548,2.0191321082,-0.196666533
C,0,-4.8128452903,0.8245882123,-0.2993288252
H,0,-4.6682687823,-1.3233768524,-0.2939989808
H,0,-2.2177794048,-1.352168155,0.0174243235
H,0,-4.6235493689,2.9726818985,-0.2711434104
H,0,-5.887376049,0.8382838069,-0.4484305248
C,0,-0.5052997983,0.8051020597,0.2591477327
H,0,-0.0330842575,0.5939929075,-0.713970573
H,0,-0.192206142,-0.0159225934,0.9136168432
Be,0,0.0192775511,2.3651208092,0.7660677669
S,0,-1.7913277869,3.5099627514,0.152603604
H,0,-2.1832054181,3.8852689932,1.3851230751
Cl,0,1.3671718761,3.3412864897,1.5866116362

1_SH_BeCl_2
0,1
C,0,-4.197512601,-0.279311881,0.0069599684
C,0,-3.6456671815,0.1848390387,-1.1839722533
C,0,-2.7778489576,1.2839142633,-1.2309335437
C,0,-2.5004001486,1.9358469954,-0.0122682354
C,0,-3.050940544,1.4735730051,1.1863919928
C,0,-3.8894744967,0.363327604,1.2022624586
H,0,-4.8595367909,-1.1392664456,-0.0020345258
H,0,-3.8958353481,-0.3303119881,-2.1110959072
H,0,-2.8283588944,2.00241212,2.108100466
H,0,-4.3089951412,0.0156368833,2.14069877
C,0,-2.1861348909,1.7432967991,-2.5445648447
H,0,-2.7707024451,2.5872878368,-2.9440836261
S,0,-1.3937012886,3.3346855079,0.0988480011
H,0,-1.8915397436,4.020593352,-0.9429059371
H,0,-1.1815464219,2.1513839056,-2.3690828068
Be,0,-2.0667269675,0.5454652453,-3.7231149206
Cl,0,-1.9302731384,-0.7157532418,-5.0051990562

1_PH2_BeH_1
0,1
C,0,-4.3488571973,-0.2514667971,-0.7347121186
C,0,-2.9732923421,-0.4313893484,-0.8271818992

C,0,-2.0761197236,0.5377326197,-0.352418523
C,0,-2.6293742693,1.695685467,0.2299983394
C,0,-4.0127839145,1.887257864,0.3135359941
C,0,-4.8770770916,0.9124325833,-0.1698955081
H,0,-5.0179823993,-1.0218643589,-1.1067372778
H,0,-2.5752600338,-1.3377669438,-1.2755560389
H,0,-4.4120115555,2.7902579621,0.7680737372
H,0,-5.9510435276,1.0510697693,-0.0996166502
C,0,-0.5796326746,0.3850729266,-0.4489790524
H,0,-0.3072274755,-0.6760972257,-0.4839405942
P,0,-1.3846785258,2.8209436129,0.9313240709
H,0,-1.3664432119,3.9304116441,0.0598192321
H,0,-2.0546059589,3.4309201898,2.0098178448
H,0,-0.2515308074,0.7951091871,-1.4206156182
Be,0,0.3348331141,1.2910426278,0.7287876972
H,0,1.5031614747,1.4208114503,1.3795434648

1_PH2_BeH_2
0,1
C,0,-4.0134468728,-0.3554702483,0.0664511406
C,0,-2.6228160144,-0.3114753433,0.051911513
C,0,-1.9215892011,0.9024395454,-0.0052357765
C,0,-2.6743012118,2.0998050079,-0.0350460013
C,0,-4.0735528814,2.0412332108,-0.0257107634
C,0,-4.7497679624,0.8256080151,0.0247023196
H,0,-4.5199405849,-1.3153265697,0.1052310209
H,0,-2.0580413403,-1.2402771602,0.0742620124
H,0,-4.6391420059,2.969183643,-0.0548839829
H,0,-5.8345280143,0.8031962534,0.0318582816
C,0,-0.4184436727,0.9015965992,0.025442055
H,0,-0.0019693338,1.5610152076,-0.7457236005
H,0,-0.0383013568,-0.1074706405,-0.1676365373
Be,0,0.0417112147,1.4532153182,1.5602725232
P,0,-1.946260089,3.7940480635,-0.094162749
H,0,-0.9596658158,3.6364262768,0.921716789
H,0,-1.0066375891,3.5835924125,-1.1370920562
H,0,0.3757976217,1.8404620487,2.7869808617

1_PH2_BeF_1
0,1
C,0,-4.1092512863,-0.3787375873,-0.1275797813
C,0,-2.7228659631,-0.3986338294,-0.0252295572

C,0,-1.9793562718,0.7874550121,0.0681523702
C,0,-2.6973762989,2.0016215369,0.0660189049
C,0,-4.0919781836,2.0248324231,-0.0467217367
C,0,-4.8017287531,0.8343106806,-0.1452904156
H,0,-4.6573395521,-1.3138650997,-0.1965437827
H,0,-2.1947862287,-1.3483793989,-0.0192916135
H,0,-4.6224189875,2.9734817674,-0.0451628464
H,0,-5.8837354701,0.8486784797,-0.2263285387
C,0,-0.4765393433,0.7942158984,0.1668264081
H,0,-0.0581108938,0.8811164215,-0.8495487648
H,0,-0.1151106837,-0.166353282,0.5501578467
Be,0,0.1542089475,2.1564728613,1.0455709325
P,0,-1.6723551361,3.4807920035,0.3369588146
H,0,-1.5679708522,4.0715004963,-0.9393193019
H,0,-2.5769391629,4.3985732613,0.90394106
F,0,1.1850407497,2.6846856552,1.8426953916

1_PH2_BeF_2
0,1
C,0,-4.0329287613,-0.3591278224,0.0367744384
C,0,-2.6416809073,-0.318443274,0.0530010819
C,0,-1.9386182661,0.8936026975,0.0241180217
C,0,-2.6853340679,2.0931252195,-0.0185981025
C,0,-4.0842625947,2.038521578,-0.0436664078
C,0,-4.764378085,0.8239507371,-0.014156672
H,0,-4.5424653736,-1.3177800404,0.0581191129
H,0,-2.0799714184,-1.2487584684,0.0821070649
H,0,-4.6467005303,2.9679095836,-0.0838321774
H,0,-5.8491314569,0.8047559181,-0.0318817753
C,0,-0.4348182358,0.8853091188,0.0722815071
H,0,-0.0135430093,1.4720187137,-0.7543350902
H,0,-0.0628794823,-0.1380544177,-0.0440724494
Be,0,0.1276731588,1.5648987611,1.5193561043
P,0,-1.9504415372,3.7852964103,-0.0419988106
H,0,-1.0667872489,3.647485723,1.069067326
H,0,-0.9165864956,3.5578301497,-0.9859210245
F,0,0.6151532318,2.0692964526,2.7108162825

1_PH2_BeCl_1
0,1
C,0,-4.1155469448,-0.3828729907,-0.1862613771
C,0,-2.734723826,-0.4025641074,-0.0289503229

C,0,-1.9958600491,0.7833778087,0.1024725525
C,0,-2.7166562804,1.9948287708,0.0800617752
C,0,-4.1062708282,2.0199516184,-0.0877216835
C,0,-4.8091513047,0.8298079482,-0.222958377
H,0,-4.6591226123,-1.3181636655,-0.2833786361
H,0,-2.2062060861,-1.351876163,-0.0083947081
H,0,-4.6374116654,2.9681898439,-0.1005074582
H,0,-5.8870211337,0.8433627924,-0.3475671675
C,0,-0.4944377454,0.7910683075,0.2552333901
H,0,-0.0430604742,0.7547371029,-0.7505312029
H,0,-0.1584397977,-0.1262975997,0.7519751659
Be,0,0.1263755331,2.233047048,0.9929852979
Cl,0,1.5899481518,2.9339223856,1.887524269
P,0,-1.6974498886,3.4677187275,0.3842101413
H,0,-1.6614385752,4.1595408306,-0.8426268054
H,0,-2.5456771631,4.3390404719,1.0915082669

1_PH2_BeCl_2
0,1
C,0,-4.0146230442,-0.3565230492,0.0741296962
C,0,-2.623957615,-0.3029897375,0.0801245644
C,0,-1.93180944,0.9144369878,0.0196476594
C,0,-2.6890770291,2.106344967,-0.0413782625
C,0,-4.0880977217,2.0379350537,-0.0548593846
C,0,-4.7570293019,0.8186998773,0.0036294684
H,0,-4.5156734207,-1.3189772411,0.1193222099
H,0,-2.0538654473,-1.2276330266,0.126104542
H,0,-4.6591093124,2.961412249,-0.108971802
H,0,-5.8416465296,0.7885891166,-0.0055530014
C,0,-0.427427669,0.917138311,0.0615616087
H,0,-0.009084621,1.54080857,-0.7382443888
H,0,-0.0486491791,-0.098789065,-0.0961829755
Be,0,0.1101043378,1.5146747771,1.5477093788
Cl,0,0.711440046,2.0606665906,3.1597849975
P,0,-1.9735054771,3.8060830184,-0.1053860175
H,0,-1.0507839308,3.6881060663,0.9739419114
H,0,-0.968443045,3.5769054046,-1.0804340443

1_COOH_BeH_1
0,1
C,0,-4.1131575683,-0.3428215982,-0.0900521316
C,0,-2.7357245187,-0.3769532771,0.0525456936

C,0,-1.9626043715,0.7995771241,0.0940430501
C,0,-2.6812096256,2.0212373688,0.0330844508
C,0,-4.0805194812,2.0544842217,-0.1379726422
C,0,-4.7974577084,0.8765585917,-0.2055622984
H,0,-4.6696825669,-1.2751606811,-0.1261276619
H,0,-2.2207908752,-1.3310108433,0.1149743858
H,0,-4.5863823392,3.0123353544,-0.1917997192
H,0,-5.8744608924,0.8950941571,-0.3299729001
C,0,-0.488679025,0.7671612901,0.1955665232
H,0,-0.0732422592,1.0397189263,-0.7953441817
H,0,-0.1260036408,-0.2390829956,0.4112653335
Be,0,0.2350653225,2.1509163688,0.967733495
C,0,-1.9824564639,3.2716727757,0.2788417697
O,0,-0.8119035872,3.3980372959,0.6988235305
O,0,-2.681420424,4.3815292314,0.0919854442
H,0,-2.1216827947,5.1336977049,0.3441072119
H,0,1.4267211595,2.4613564845,1.525541287

1_COOH_BeH_2
0,1
C,0,-4.1048850532,-0.3532064394,-0.049127922
C,0,-2.7208052347,-0.378618012,0.0538477603
C,0,-1.9471794176,0.7948593444,0.0552863305
C,0,-2.6644153229,2.0118474241,-0.017927452
C,0,-4.0636930923,2.0391124547,-0.1525373813
C,0,-4.7885425867,0.8611992059,-0.168455607
H,0,-4.6583774569,-1.2878289102,-0.0486514532
H,0,-2.2038319413,-1.3316469718,0.119384843
H,0,-4.5586317919,3.002574613,-0.2208391717
H,0,-5.8689144016,0.8823866723,-0.2599061611
C,0,-0.4545072699,0.729741915,0.1051008338
H,0,-0.0813208675,0.8651686571,-0.9274206669
H,0,-0.1311894211,-0.2711792378,0.4015084558
Be,0,0.3245896962,2.0643018629,0.8664868352
H,0,1.4718938733,2.4952292722,1.4335329741
C,0,-2.0454105943,3.3402957,0.1033830083
O,0,-2.5365757264,4.4116909514,-0.1160492761
O,0,-0.7338439703,3.3369761634,0.6176398624
H,0,-0.4511618807,4.2552894847,0.762130878

1_COOH_BeF_1
0,1

C,0,-4.0964019207,-0.3404985307,-0.0931160877
C,0,-2.7172015311,-0.3676454559,0.0451027581
C,0,-1.950678606,0.8106450718,0.0979214426
C,0,-2.674407972,2.0291420304,0.0469659417
C,0,-4.0735220055,2.0566018335,-0.1200246975
C,0,-4.7859030588,0.8754684126,-0.1943356868
H,0,-4.6478860869,-1.2755344842,-0.1359260763
H,0,-2.1973089965,-1.3195900643,0.0975228506
H,0,-4.5849731201,3.0117899644,-0.1656407772
H,0,-5.8633148496,0.8905000159,-0.3151697597
C,0,-0.4681710317,0.776622634,0.1920208435
H,0,-0.0674985852,0.9796035656,-0.818820679
H,0,-0.1194313397,-0.222425046,0.4605986585
Be,0,0.2115928288,2.1644955772,0.9893990012
C,0,-1.9828425271,3.2902528165,0.2826263019
O,0,-0.8101843656,3.4268581061,0.6874483299
O,0,-2.6938821437,4.3911488129,0.0948927761
H,0,-2.1382752778,5.1518269689,0.3307102615
F,0,1.4078929593,2.5091206714,1.6733466184

1_COOH_BeF_2
0,1
C,0,-4.0942504988,-0.3509228492,-0.058764685
C,0,-2.711138355,-0.3713750357,0.0585924778
C,0,-1.9409589748,0.8036236685,0.0753743088
C,0,-2.6594925185,2.0196153242,-0.0024483362
C,0,-4.0577235913,2.0416482792,-0.1501435158
C,0,-4.7792037661,0.8617387674,-0.1789416117
H,0,-4.6450195227,-1.2870976609,-0.069119459
H,0,-2.1919672613,-1.3230944562,0.1246356046
H,0,-4.5553015213,3.0035165978,-0.2196874742
H,0,-5.8585411127,0.8807432172,-0.2820148235
C,0,-0.4439848882,0.7265958958,0.1356957377
H,0,-0.0702434759,0.7944801416,-0.9014252834
H,0,-0.1380838179,-0.2611461463,0.4898777525
Be,0,0.3146247593,2.0798620612,0.8757274524
C,0,-2.0518467179,3.3565751656,0.1217741538
O,0,-2.5605148751,4.4212321911,-0.0892208189
O,0,-0.7368975481,3.36477754,0.6160281038
H,0,-0.4485086749,4.283265859,0.7563971561
F,0,1.5054413611,2.5641894396,1.4788922601

1_COOH_BeCl_1
0,1
C,0,-4.0977808402,-0.341478444,-0.120451016
C,0,-2.7222652077,-0.3637514645,0.0532690372
C,0,-1.9626014389,0.8174226429,0.1212539113
C,0,-2.6883918062,2.0330427393,0.0480743655
C,0,-4.0833066514,2.0558665709,-0.1541061241
C,0,-4.7888024642,0.871919494,-0.2426872481
H,0,-4.643772967,-1.278986967,-0.1737655479
H,0,-2.2005369267,-1.3135738328,0.1227649
H,0,-4.5968910624,3.0089787815,-0.216017023
H,0,-5.8628090125,0.8832321302,-0.3911359599
C,0,-0.4814536114,0.7933330812,0.2514141037
H,0,-0.0578526928,0.9651761593,-0.7562713246
H,0,-0.1322240781,-0.1941194089,0.559127491
Be,0,0.1703337137,2.1970125118,1.0234737218
C,0,-2.007942495,3.2959850209,0.294802188
O,0,-0.8435684485,3.4400541656,0.7303298229
O,0,-2.7129402664,4.3942106619,0.0860908918
H,0,-2.1680341263,5.1571991787,0.3399214187
Cl,0,1.7649053822,2.6219119789,1.9072023918

1_COOH_BeCl_2
0,1
C,0,-2.8585440104,2.9987796934,-4.0498021935
C,0,-1.5238166928,3.0187406507,-3.6682180471
C,0,-1.1258011444,2.9067104919,-2.3258906716
C,0,-2.1660735415,2.8033917299,-1.3741314225
C,0,-3.5175895605,2.7556161442,-1.7602158175
C,0,-3.8688197299,2.8523510128,-3.0944593509
H,0,-3.1151803966,3.0870255814,-5.1013972928
H,0,-0.7497301901,3.1086779618,-4.4248113484
H,0,-4.2741204141,2.6616875933,-0.9878407849
H,0,-4.9118674242,2.8249406662,-3.389889102
C,0,-1.9631278577,2.7969705435,0.0837333701
O,0,-2.7623437272,2.5616411647,0.9433762274
O,0,-0.6741908393,3.1903092282,0.5096953968
H,0,-0.6606627877,3.2591128287,1.4809869485
C,0,0.3299433983,2.8659759996,-1.9656479049
H,0,0.9270225429,3.2812211919,-2.7815180699
H,0,0.6241549228,1.8031730649,-1.9010709237
Be,0,0.6936359608,3.4348033914,-0.3939606146

Cl,0,2.0958538317,4.0461566514,0.6800030715

1_CONH2_BeH_1
0,1
C,0,-4.3478867248,-0.2552494586,0.4891449626
C,0,-2.9862380356,-0.3991532762,0.7059914778
C,0,-2.0710725672,0.6355762811,0.4309561625
C,0,-2.6157509643,1.829420433,-0.1016282668
C,0,-3.9975386972,1.9748926325,-0.3142178841
C,0,-4.869152022,0.941109077,-0.0168220068
H,0,-5.0171921986,-1.080085082,0.7163305186
H,0,-2.5990057185,-1.3307152923,1.1082301194
H,0,-4.3911128674,2.8795206597,-0.7705953343
H,0,-5.9329345466,1.0504697137,-0.1961072271
C,0,-0.6162573209,0.4805720836,0.6512114957
H,0,-0.3069108836,1.1332148478,1.4881504955
C,0,-1.6965050913,2.891768526,-0.548213439
O,0,-0.5089679113,2.6738692205,-0.9139321542
N,0,-2.1371053319,4.1525100484,-0.6259838569
H,0,-1.5004730598,4.8578012704,-0.9685604756
H,0,-3.0321727896,4.4263938031,-0.2558472102
H,0,-0.3648339254,-0.5422699919,0.9361921013
Be,0,0.3190573196,1.3036482954,-0.582863162
H,0,1.5375586566,1.1544162889,-1.1566159365

1_CONH2_BeH_2
0,1
C,0,-3.9906773178,-0.268636234,-0.5959463749
C,0,-2.6365255851,-0.3338339434,-0.2871780289
C,0,-1.9366155542,0.7764567425,0.2113051674
C,0,-2.6757127972,1.9725071633,0.3467988295
C,0,-4.0516280593,2.0305987085,0.0854736444
C,0,-4.7138436413,0.9107228244,-0.3941925607
H,0,-4.4948053844,-1.1517609166,-0.9777924241
H,0,-2.1006287793,-1.2712585616,-0.4083073877
H,0,-4.5766560432,2.9679536647,0.2452544573
H,0,-5.7746878467,0.9530423985,-0.6158911382
C,0,-0.5071566117,0.6941265632,0.6486335863
H,0,-0.0539957223,-0.2399667583,0.3044496501
H,0,-0.5015156732,0.6311900487,1.7516547512
Be,0,0.4405376023,2.1212354374,0.3718524666
C,0,-2.0213971887,3.2396335609,0.7379084319

O,0,-2.4730196584,4.092407694,1.4589863817
N,0,-0.7119317599,3.4541457878,0.126653271
H,0,-0.8016379452,3.4303813982,-0.8927777568
H,0,-0.3611844042,4.3726685577,0.3981437202
H,0,1.7327573697,2.511127864,0.2623293137

1_CONH2_BeF_1
0,1
C,0,-4.0725394676,-0.3089724579,-0.1470663662
C,0,-2.6885185889,-0.3313576203,-0.0602094414
C,0,-1.9253295306,0.845992136,0.0469012713
C,0,-2.6487304088,2.063044692,0.0791065993
C,0,-4.0507264472,2.0830929487,-0.0136926502
C,0,-4.7680145988,0.9045654861,-0.1313871047
H,0,-4.6203899741,-1.2434067396,-0.2284518189
H,0,-2.1617745512,-1.2808964889,-0.0816345364
H,0,-4.5941857794,3.0208626226,0.0665366299
H,0,-5.8508354827,0.9246620935,-0.186542679
C,0,-0.4430722619,0.8153961803,0.1458407517
H,0,-0.0199730726,1.171860921,-0.8097886226
H,0,-0.0840566847,-0.2057099266,0.2868484014
Be,0,0.132312839,2.0861793668,1.200472909
C,0,-1.9226887895,3.326335595,0.3224754088
O,0,-0.8152398088,3.3864918132,0.9203340189
N,0,-2.4721363761,4.483487816,-0.0630056962
H,0,-1.9716713772,5.3368107727,0.1410007693
H,0,-3.2975605292,4.5157200306,-0.6380372746
F,0,1.2319489606,2.2851549089,2.0831781004

1_CONH2_BeF_2
0,1
C,0,-3.9836243046,-0.2679046896,-0.5893221036
C,0,-2.6312643148,-0.3300809126,-0.2718461233
C,0,-1.9319187482,0.7837301512,0.2179626141
C,0,-2.6712427465,1.9811183041,0.3420020969
C,0,-4.0458316137,2.0363869571,0.073229455
C,0,-4.7061716322,0.9133302816,-0.4020690146
H,0,-4.4863996838,-1.1537687053,-0.9666449875
H,0,-2.0954416376,-1.2689975875,-0.3806847967
H,0,-4.5718852598,2.9745639786,0.2243775613
H,0,-5.7659301212,0.9547608501,-0.6293615686
C,0,-0.5007786608,0.6882656974,0.6646979732

H,0,-0.0400057109,-0.2184878363,0.2607035412
H,0,-0.5085313414,0.5455675553,1.7587607127
Be,0,0.4276429295,2.1311729785,0.429473337
C,0,-2.0274247568,3.2546589897,0.7396771723
O,0,-2.4945866969,4.1009760498,1.4573447133
N,0,-0.713192397,3.4772048774,0.1446676687
H,0,-0.7933377509,3.4680354467,-0.8752479509
H,0,-0.3569333908,4.3884130268,0.4355432259
F,0,1.7797285182,2.573831937,0.3679379135

1_CONH2_BeCl_1
0,1
C,0,-4.1755409694,-0.1535221328,-0.9207652636
C,0,-2.8514666093,-0.5110886039,-0.7129423659
C,0,-1.8989305354,0.4000519145,-0.2220304177
C,0,-2.3659340891,1.7047662693,0.067492812
C,0,-3.7073092155,2.0659209634,-0.1473583805
C,0,-4.6144514451,1.1458280134,-0.6443385923
H,0,-4.8768680536,-0.89054721,-1.3006391381
H,0,-2.5228062985,-1.5212641349,-0.938649867
H,0,-4.0631977892,3.0547105549,0.129804226
H,0,-5.6517138537,1.4246585596,-0.7928025477
C,0,-0.4801921169,0.0118293857,0.0024139331
H,0,-0.3565895407,-1.0702378065,-0.0704962903
C,0,-1.4511203024,2.67270629,0.7023970352
O,0,-0.4485673146,2.3346209412,1.3932297277
N,0,-1.6979435745,3.9808206561,0.5970393212
H,0,-2.4134710064,4.3411028093,-0.0124084249
H,0,-1.0763810263,4.6210442,1.0709064647
H,0,0.1354056843,0.4530036476,-0.8016748537
Be,0,0.1814183958,0.8500460517,1.372518981
Cl,0,1.5138310305,0.4741511715,2.641735631

1_CONH2_BeCl_2
0,1
C,0,-3.2085227245,2.0127229033,-3.8792501452
C,0,-1.8571736082,2.3068821463,-3.734556417
C,0,-1.3373647752,2.8288956503,-2.5404393837
C,0,-2.2558723277,3.0110238057,-1.483366156
C,0,-3.6270061604,2.7640321227,-1.6398779568
C,0,-4.1073947447,2.255134664,-2.836944436
H,0,-3.5692981464,1.6084871274,-4.8204803811

H,0,-1.1795220275,2.1528687469,-4.5696874652
H,0,-4.2936609932,2.9573828311,-0.8044666793
H,0,-5.164116882,2.0430550894,-2.9582649251
C,0,-1.8159722865,3.4458530219,-0.1394004533
O,0,-2.3989931371,4.1909841289,0.6036690898
N,0,-0.5577321601,2.8409380796,0.3132451738
H,0,-0.6714509755,1.8241796018,0.3606520189
H,0,-0.3430947279,3.1776903943,1.253564128
C,0,0.0993462825,3.2459218192,-2.411957873
H,0,0.1432695128,4.3377002202,-2.565187174
H,0,0.6950399145,2.8154588838,-3.2224469505
Be,0,0.7622376089,3.0074452094,-0.8397163491
Cl,0,2.4484049281,2.8166085738,-0.0430659753

TWO CARBON

2_OH_BeH_1
0,1
C,0,-1.5838674428,-0.291267531,-0.0055819329
C,0,-0.1968581227,-0.4074803631,0.0811589588
C,0,0.6454973802,0.7053363214,0.0269218158
C,0,0.0142885158,1.9434959949,-0.1043935452
C,0,-1.3602581111,2.0980976917,-0.1900165192
C,0,-2.1686952884,0.9636907624,-0.1447939305
H,0,-2.2054941753,-1.179493741,0.0377240654
H,0,0.2568028671,-1.3885658789,0.1924658983
H,0,-1.7978018097,3.0887357311,-0.2878807341
H,0,-3.2462654277,1.0684175849,-0.2112930781
O,0,0.8422484218,3.0763307671,-0.1483742164
H,0,0.355924852,3.896882334,-0.3029871261
C,0,2.1509578959,0.5972045857,0.1018977697
H,0,2.5516216938,0.7870168748,-0.9048029399
H,0,2.3964042882,-0.4477869753,0.3307724178
C,0,2.7735742534,1.600503465,1.0953016133
H,0,3.8640622488,1.4823290465,1.0860633619
H,0,2.457877973,1.3271901602,2.1142893883
Be,0,2.2872577654,3.2145495506,0.7255554337
H,0,2.5754477223,4.5375302789,0.8401291795

2_OH_BeH_2
0,1

C,0,-1.5957499856,-0.2072942716,-0.1994171843
C,0,-0.2033850745,-0.2259463331,-0.2161348719
C,0,0.5521656375,0.9220363294,0.0425990647
C,0,-0.1386285017,2.1100938413,0.3255657458
C,0,-1.532567958,2.1424218189,0.3515257005
C,0,-2.2577596936,0.9860147605,0.0865517418
H,0,-2.1561389825,-1.1131576938,-0.4042409007
H,0,0.3205202318,-1.1555149178,-0.4311707373
H,0,-2.0232317701,3.0831626205,0.578644939
H,0,-3.3425648236,1.0186477168,0.1052835031
O,0,0.5071400375,3.2843185097,0.5857120165
H,0,1.4617096709,3.1494588935,0.5868828683
C,0,2.0598770024,0.9062807581,-0.0621389874
H,0,2.5115468679,1.4236598753,0.7985493933
H,0,2.3998224998,-0.131187499,0.0224742278
C,0,2.5412366467,1.5054609853,-1.406739063
H,0,3.6346583782,1.4337073573,-1.4810853325
H,0,2.3131417451,2.5830252009,-1.4341293494
Be,0,1.7636409705,0.8045304322,-2.7400052912
H,0,1.2056969312,0.2480810756,-3.8116524031

2_OH_BeF_1
0,1
C,0,-1.8491894602,0.1250579399,1.1607781144
C,0,-0.4851438915,-0.0964255822,1.3497656869
C,0,0.4766772305,0.4054614466,0.4702914847
C,0,-0.0105671359,1.1321263836,-0.6186844752
C,0,-1.3577157391,1.3699822955,-0.8398193551
C,0,-2.288043251,0.8620636038,0.0652616148
H,0,-2.5657488166,-0.2805466815,1.8672291737
H,0,-0.1463341299,-0.6743720662,2.2052503733
H,0,-1.6806690238,1.9411828618,-1.7068177173
H,0,-3.3457321459,1.0403791473,-0.0957128573
O,0,0.9407929978,1.6353817768,-1.5177053416
H,0,0.5557083081,2.1469025536,-2.2437233452
C,0,1.9597955297,0.1900047621,0.6722550053
H,0,2.3961735767,1.1461489109,0.9964836512
H,0,2.0819992135,-0.4976705743,1.5182865396
C,0,2.6842582498,-0.2923193086,-0.6036967776
H,0,3.7562067162,-0.3833218875,-0.391325577
H,0,2.3413670638,-1.310286614,-0.8404306633
Be,0,2.3617661995,0.7832286338,-1.909624224

F,0,2.7741024584,1.2691577886,-3.1872191704

2_OH_BeF_2
0,1
C,0,-1.5915492885,-0.1981886183,-0.1848687724
C,0,-0.1966765906,-0.2294915056,-0.2071957393
C,0,0.569628292,0.921467547,-0.0194652498
C,0,-0.1100047088,2.1308636952,0.1910529767
C,0,-1.5017798439,2.1779596997,0.2235711179
C,0,-2.2419315216,1.0118813392,0.034413979
H,0,-2.1606913527,-1.1094207167,-0.3336277289
H,0,0.3181594483,-1.1750466356,-0.3673567325
H,0,-2.0064567911,3.1266372346,0.3930929356
H,0,-3.3261221336,1.0572247797,0.0584648043
O,0,0.6604811456,3.247841501,0.3604758203
H,0,0.0988911518,4.0123336222,0.5299320705
C,0,2.0748330769,0.9026744105,-0.1073520886
H,0,2.492684773,1.5253367434,0.6915238946
H,0,2.419784273,-0.1224199171,0.0677892776
C,0,2.5654938193,1.3995226127,-1.488639857
H,0,3.6576169568,1.3097898052,-1.5504211947
H,0,2.3465190627,2.4733485789,-1.5655819437
Be,0,1.8058246686,0.6039134066,-2.7737553866
F,0,1.2325081029,-0.0653004925,-3.8417062132

2_OH_BeCl_1
0,1
C,0,-1.5783809134,-0.2682130146,0.0926481949
C,0,-0.1910943095,-0.4061805738,0.110537324
C,0,0.6651667156,0.6960123459,0.0462602223
C,0,0.0444395315,1.944458292,-0.0213222412
C,0,-1.3291593229,2.1220360446,-0.0391256261
C,0,-2.1509427222,0.9977752771,0.0142285227
H,0,-2.2095121098,-1.1491566414,0.1425894923
H,0,0.2517650672,-1.3963816833,0.173176307
H,0,-1.755222151,3.1209661143,-0.0894070947
H,0,-3.2287295372,1.1185051615,0.0018983416
O,0,0.8845627676,3.0719571335,-0.0749222267
H,0,0.411104477,3.9089017102,-0.1934482519
C,0,2.1712144933,0.5609279358,0.0410705811
H,0,2.521992926,0.7593673346,-0.9821236255
H,0,2.410472026,-0.4906202001,0.2420728274

C,0,2.8714666267,1.5322842949,1.0162363449
H,0,3.9572073254,1.4119229701,0.9255366583
H,0,2.6253083262,1.2376948446,2.047182751
Be,0,2.3610401878,3.1393359891,0.7063239037
Cl,0,2.8302769159,4.951875115,0.8943325049

2_OH_BeCl_2
0,1
C,0,-1.585064021,-0.2075823044,-0.1772809622
C,0,-0.1905424981,-0.2382541303,-0.2141065652
C,0,0.5776834591,0.9112796394,-0.0226266955
C,0,-0.1005737211,2.1173597684,0.2101475045
C,0,-1.4921009992,2.1639045308,0.2557744066
C,0,-2.2339015742,0.9998055933,0.0604716369
H,0,-2.1551750494,-1.1174924325,-0.3305568747
H,0,0.3219597866,-1.1814593337,-0.3935946878
H,0,-1.9946286674,3.110272795,0.4429413037
H,0,-3.3178307232,1.0439331886,0.0946956207
O,0,0.6706867132,3.232204025,0.3882623403
H,0,0.1097349053,3.9949954917,0.567373482
C,0,2.0819786226,0.8961715686,-0.1315821737
H,0,2.5129348948,1.4909202478,0.6815389072
H,0,2.4289089829,-0.1343774222,0.0026862991
C,0,2.5507390375,1.4386152044,-1.5048756723
H,0,3.639545028,1.333987148,-1.5967218779
H,0,2.3487974253,2.5187060148,-1.5336803939
Be,0,1.7343807423,0.7123824196,-2.7885464479
Cl,0,0.9036924962,-0.0860984822,-4.1836782199

2_NH2_BeH_1
0,1
C,0,-1.556740873,-0.2547558514,0.1886196406
C,0,-0.1725497994,-0.3791670294,0.0756093759
C,0,0.6549794705,0.738631642,-0.0434107784
C,0,0.0357944173,1.9962754231,-0.0572955562
C,0,-1.3421609273,2.1392680738,0.0588159429
C,0,-2.1447129395,1.0069493203,0.1846770867
H,0,-2.1738324324,-1.1420607301,0.288113731
H,0,0.285675135,-1.3643778819,0.0931491269
H,0,-1.7882769673,3.1309323342,0.0510237577
H,0,-3.2201922863,1.1152401274,0.2779864937
C,0,2.1603138972,0.6390246034,-0.1013149952

H,0,2.5024353763,1.0114263252,-1.0804858468
H,0,2.4361150561,-0.4228702606,-0.0803420651
C,0,2.8190369966,1.472692075,1.0215097253
H,0,3.9106925965,1.4394389815,0.9054059046
H,0,2.617554066,0.9753735044,1.9826929242
Be,0,2.206975281,3.0959066646,1.0684295264
N,0,0.8902926549,3.166799975,-0.1479476539
H,0,0.3520024192,4.0263561382,-0.066497762
H,0,1.3611695364,3.1913460666,-1.0553664436
H,0,2.4128755822,4.2983327589,1.6733245653

2_NH2_BeH_2
0,1
C,0,-1.5444936327,-0.251367167,-0.1149819571
C,0,-0.1525159889,-0.2573033913,-0.1775414313
C,0,0.6031815867,0.9057586857,-0.0110465414
C,0,-0.074555692,2.1172775256,0.2356778006
C,0,-1.4737519273,2.1239493882,0.3025081515
C,0,-2.2019766504,0.9526815484,0.1263972458
H,0,-2.1040301701,-1.1705577702,-0.2507300051
H,0,0.37232696,-1.193485277,-0.3593025212
H,0,-1.989023814,3.0633878412,0.4877896728
H,0,-3.2858662614,0.984177074,0.1804326139
C,0,2.1059809681,0.8768211916,-0.1684023675
N,0,0.6334920472,3.3169635991,0.3564670623
H,0,0.1398737301,4.0509660125,0.8448894387
H,0,1.5891749563,3.2300054337,0.6739563784
H,0,2.5887218979,1.4081045041,0.666195789
H,0,2.4402710434,-0.1627576312,-0.0811387666
C,0,2.5458449749,1.4546933042,-1.5356772774
H,0,3.6322458904,1.3497410468,-1.6575478401
H,0,2.3436296551,2.5366147529,-1.5482242867
Be,0,1.6768120859,0.7844206233,-2.8242029532
H,0,1.040814711,0.2602053355,-3.8697147753

2_NH2_BeF_1
0,1
C,0,-1.5749853055,-0.2679907945,0.1243190258
C,0,-0.1840588711,-0.3469910328,0.0712872972
C,0,0.6114650133,0.7957862897,-0.0345600901
C,0,-0.0485138094,2.0314715354,-0.0978981375
C,0,-1.4343331531,2.1284440499,-0.0437841887

C,0,-2.204010573,0.9724980329,0.0710715747
H,0,-2.1650080582,-1.1743199964,0.2148209497
H,0,0.3048830575,-1.3159167694,0.1263015685
H,0,-1.9122627854,3.1040441414,-0.0902315017
H,0,-3.2854628402,1.0462013155,0.1167471756
C,0,2.1216222901,0.7332691931,-0.0352477277
H,0,2.4895790197,1.0950141155,-1.0084766862
H,0,2.4196870347,-0.3212374915,0.0167501387
C,0,2.7301224771,1.5961526298,1.094633239
H,0,3.8244357366,1.5813662059,1.0107432567
H,0,2.503617418,1.1150134925,2.0571818484
Be,0,2.0778295702,3.2004752076,1.0497604311
N,0,0.7685346473,3.2305156148,-0.1797324045
H,0,1.2164305606,3.2931321391,-1.0961549265
H,0,0.2099254977,4.0746498199,-0.0623284108
F,0,2.2369631929,4.4998455514,1.6274041284

2_NH2_BeF_2
0,1
C,0,-1.5563076927,-0.2377246306,-0.143772693
C,0,-0.1647389653,-0.2461293701,-0.2124543522
C,0,0.594200586,0.9111126007,-0.0231573396
C,0,-0.0783031896,2.1200068566,0.2498282113
C,0,-1.4766250022,2.1283925417,0.3242210403
C,0,-2.208608854,0.9627350894,0.1269127521
H,0,-2.1190558187,-1.1520620276,-0.2971657022
H,0,0.3574082716,-1.179500545,-0.4158272064
H,0,-1.9896135012,3.0644855723,0.531294899
H,0,-3.2921587693,0.9967069524,0.1866100642
C,0,2.0948870438,0.8785959045,-0.1904729322
N,0,0.6345973746,3.3155362592,0.386360854
H,0,0.1405921422,4.0461340595,0.8794232357
H,0,1.5867379143,3.2211313959,0.7122906821
H,0,2.5872115172,1.4067418576,0.6399187658
H,0,2.4281897219,-0.1617694521,-0.1102516109
C,0,2.5277478289,1.4647307196,-1.5555180621
H,0,3.6119312242,1.3612434204,-1.6878890801
H,0,2.316940018,2.5430968179,-1.5682897206
Be,0,1.6626643806,0.767360348,-2.8333083288
F,0,1.0216081998,0.1821960497,-3.9112327665

2_NH2_BeCl_1

0,1
C,0,-1.5887584922,-0.2713730124,-0.1074509419
C,0,-0.1988823937,-0.3603497948,-0.0487288735
C,0,0.6065035201,0.7788041326,0.0153109818
C,0,-0.0448466143,2.019760144,0.0312921056
C,0,-1.4293015301,2.1280570191,-0.0304904533
C,0,-2.2081858689,0.9752548223,-0.1040401531
H,0,-2.1863416121,-1.1754732252,-0.1652320746
H,0,0.2812146572,-1.3350124814,-0.0656499833
H,0,-1.8980028508,3.1091195518,-0.0231450727
H,0,-3.2886432363,1.0554148347,-0.1589069344
C,0,2.115975405,0.7044581009,0.0243904679
N,0,0.7790460287,3.2200949723,0.0811395996
H,0,0.2197053296,4.0615460737,-0.0567267351
H,0,1.2174403501,3.3082701398,1.0013869543
H,0,2.4821006469,1.0744677227,0.9951365804
H,0,2.4061075247,-0.3526158358,-0.0144591591
C,0,2.7379429892,1.54886915,-1.1123819721
H,0,3.8314572651,1.5304139284,-1.0238465972
H,0,2.5136644267,1.0597572302,-2.0715603339
Be,0,2.0975459406,3.1470891813,-1.0927791658
Cl,0,2.4141302148,4.8344702359,-1.8746724698

2_NH2_BeCl_2
0,1
C,0,-1.6026454238,-0.154654255,-0.27767755
C,0,-0.2097577886,-0.1908695709,-0.29008327
C,0,0.5635748633,0.932199423,0.0119113963
C,0,-0.093133408,2.1368104053,0.3349486095
C,0,-1.4932664988,2.1714850714,0.3572181129
C,0,-2.2403538649,1.0389517302,0.052645267
H,0,-2.1767063627,-1.0429535332,-0.5175500349
H,0,0.2994472617,-1.122819714,-0.5325185702
H,0,-1.9945922634,3.1003977966,0.6180079914
H,0,-3.3245050788,1.0927881788,0.0731128609
C,0,2.0697667162,0.8746802719,-0.0815396682
N,0,0.6387598211,3.270151828,0.6983549169
H,0,1.5646644743,3.3481832537,0.3035116747
H,0,0.1294374899,4.1406530382,0.6467776889
H,0,2.5198856385,1.4378394257,0.7465628204
H,0,2.388413265,-0.1645077972,0.0547606973
C,0,2.5700257804,1.387717031,-1.4563798832

H,0,3.659953637,1.2838837244,-1.5314288371
H,0,2.3674574245,2.4662924526,-1.5478794901
Be,0,1.7594501893,0.6200113207,-2.7245001979
Cl,0,0.9805382178,-0.2145883911,-4.1264335146

2_SH_BeH_1
0,1
C,0,-1.5131592744,-0.511028853,0.3168370624
C,0,-0.1250645007,-0.4139168979,0.2718258271
C,0,0.5211562958,0.807889213,0.0587319219
C,0,-0.2921612213,1.9377759033,-0.1066204265
C,0,-1.6831876356,1.8615899973,-0.0578058531
C,0,-2.2958035501,0.6292866142,0.149447554
H,0,-1.9838081839,-1.4751305939,0.4803402169
H,0,0.4844215882,-1.3048280409,0.3977805936
H,0,-2.2870802958,2.7566901736,-0.1777392621
H,0,-3.3784166385,0.5651797865,0.181291176
C,0,2.0265690318,0.8965289463,-0.0313436338
S,0,0.532036642,3.5127206751,-0.3529331934
H,0,-0.4487131363,4.1324584947,-1.0254160307
H,0,2.3847717919,1.6147388373,0.7211064401
H,0,2.4404701466,-0.0750324155,0.2637867681
C,0,2.5028902152,1.3384735276,-1.4332848303
H,0,2.2457886773,0.548064901,-2.154143329
H,0,3.6020516019,1.3757818428,-1.4326721006
Be,0,1.901933686,2.850476645,-2.0049645433
H,0,1.9147792297,3.7764560835,-2.9877397672

2_SH_BeH_2
0,1
C,0,-1.1814393814,-0.4746673761,-0.2575973899
C,0,0.1813593955,-0.2715429821,-0.0592895848
C,0,0.7208263374,1.0054130038,0.1364201859
C,0,-0.1584232365,2.1014428791,0.1050868026
C,0,-1.5280564941,1.9018930078,-0.0955290524
C,0,-2.0415620954,0.6203054036,-0.2682025269
H,0,-1.5667123793,-1.4795951125,-0.3970875174
H,0,0.8520224873,-1.1294695222,-0.035065751
H,0,-2.1890212011,2.7630142945,-0.1270615177
H,0,-3.1070558215,0.4830207619,-0.4220829142
C,0,2.2139490137,1.1584249585,0.3004624316
S,0,0.3650539866,3.7836059779,0.4069110453

H,0,1.5196299792,3.7364607817,-0.2758510818
H,0,2.427368621,1.9794583178,0.9945606797
H,0,2.602930575,0.2484804243,0.7707695888
C,0,2.9112006821,1.3659599849,-1.0663632507
H,0,3.9969341983,1.4502181682,-0.9201238665
H,0,2.5997052712,2.3320989856,-1.4964896933
Be,0,2.5315783615,0.1757107249,-2.2104797197
H,0,2.2882277406,-0.7605650715,-3.1243147374

2_SH_BeF_1
0,1
C,0,-1.5541555518,-0.2475829657,0.2839410903
C,0,-0.1660883412,-0.3067529756,0.1984038695
C,0,0.6030960774,0.82361338,-0.0964387567
C,0,-0.086150596,2.0265635317,-0.3045363413
C,0,-1.4766669475,2.1010744356,-0.2380948697
C,0,-2.2132683145,0.9600175708,0.0651638749
H,0,-2.120774536,-1.1422712054,0.5212515407
H,0,0.3465570853,-1.2494908517,0.3698071907
H,0,-1.9835619822,3.0436924946,-0.4238637917
H,0,-3.2950019293,1.0179911654,0.1266058774
C,0,2.1118889016,0.7471302022,-0.1467208774
H,0,2.4565130695,1.1309508157,-1.1178734223
H,0,2.396672204,-0.3112772162,-0.1278253181
C,0,2.7787418946,1.5463174828,0.9946099885
H,0,3.8687109743,1.434380313,0.910994377
H,0,2.5100304807,1.0791973981,1.9525696653
Be,0,2.3807297985,3.2196126537,1.0285187008
S,0,0.8824857912,3.485676705,-0.7106267555
H,0,0.0853969645,4.3955315837,-0.1289634604
F,0,2.5396368368,4.4786146124,1.6530143882

2_SH_BeF_2
0,1
C,0,-1.1770707327,-0.459433562,-0.3312955013
C,0,0.1828948179,-0.2735352891,-0.0987481336
C,0,0.727377084,0.9946340613,0.1381711366
C,0,-0.142701542,2.098454196,0.1162179435
C,0,-1.5089027221,1.9151120038,-0.1166875442
C,0,-2.0285580333,0.641906093,-0.3327488137
H,0,-1.567009628,-1.456918681,-0.5063652386
H,0,0.8448007449,-1.138481489,-0.0797459901

H,0,-2.1617376703,2.7823759337,-0.1393409282
H,0,-3.0912684467,0.5163783506,-0.5133430621
C,0,2.2184369696,1.1321286499,0.3267188821
S,0,0.3960994871,3.766712723,0.474377499
H,0,1.4741848717,3.7794044431,-0.3255088821
H,0,2.4304403914,1.9531908069,1.0203744656
H,0,2.5939163047,0.2178900023,0.7994018888
C,0,2.9371763217,1.3410930558,-1.0289606805
H,0,4.0212951467,1.4053476214,-0.8721760562
H,0,2.6408797613,2.3101713281,-1.4581231333
Be,0,2.5440546988,0.1431831836,-2.1611438577
F,0,2.280599365,-0.8521188212,-3.0852273841

2_SH_BeCl_1
0,1
C,0,-1.503246232,-0.520209651,0.4015642317
C,0,-0.1183335917,-0.4094526798,0.3140519411
C,0,0.5084154312,0.8138209191,0.0558005836
C,0,-0.3236857551,1.9289955614,-0.1118445741
C,0,-1.7112448432,1.8410392142,-0.0170327687
C,0,-2.3037772063,0.6075457514,0.2353801519
H,0,-1.9567826606,-1.4861371572,0.5992905739
H,0,0.5040064852,-1.2909439687,0.442408124
H,0,-2.3279220299,2.7271344113,-0.1375906172
H,0,-3.3841004572,0.5329305037,0.3019352585
C,0,2.0110917308,0.9135582043,-0.0710268486
S,0,0.4637971036,3.5172639122,-0.4168084138
H,0,-0.5271280226,4.0804668984,-1.1266432337
H,0,2.3803507807,1.648483927,0.6593728147
H,0,2.4398841321,-0.048622055,0.2320051227
C,0,2.4548955746,1.3265909288,-1.492379086
H,0,2.1757663349,0.5266721107,-2.1932829479
H,0,3.5528005732,1.3655528377,-1.5210711719
Be,0,1.8230218594,2.8223167074,-2.0449902963
Cl,0,1.8645338229,4.0293190941,-3.4660105739

2_SH_BeCl_2
0,1
C,0,-1.1373545374,-0.4935965608,-0.3303569032
C,0,0.2185173922,-0.2818170753,-0.0949159967
C,0,0.7390757408,0.9964520046,0.1397995352
C,0,-0.1509435541,2.0844326234,0.1116729764

C,0,-1.5131877106,1.8750665353,-0.1232439841
C,0,-2.0090383168,0.5919238749,-0.3365554772
H,0,-1.5075294911,-1.4990548175,-0.502414909
H,0,0.8956214481,-1.1347956774,-0.071596307
H,0,-2.181326191,2.7304798521,-0.1489195887
H,0,-3.0690976533,0.4467490448,-0.517774452
C,0,2.2261757117,1.1620155325,0.3378984879
S,0,0.3557473763,3.7627984609,0.4669374118
H,0,1.4428082996,3.7875472258,-0.3200697838
H,0,2.4187600597,1.9843526651,1.035460214
H,0,2.6159111613,0.2533713344,0.8099814892
C,0,2.9500219189,1.3885425041,-1.0130296431
H,0,4.0317712168,1.4742411594,-0.8470690306
H,0,2.6400199709,2.3547067917,-1.4401600552
Be,0,2.5970144471,0.1921527739,-2.1521900996
Cl,0,2.3102269507,-1.1101841019,-3.3721017942

2_PH2_BeH_1
0,1
C,0,-1.5747716592,-0.2815707316,-0.1889556255
C,0,-0.1877179532,-0.3233838674,-0.0639697705
C,0,0.5729552925,0.8455595876,0.0297345184
C,0,-0.1104775396,2.0764763144,0.001772836
C,0,-1.5016784402,2.1213683901,-0.1240225114
C,0,-2.2371399898,0.9431581607,-0.2206995027
H,0,-2.1384873982,-1.2063546079,-0.2642254074
H,0,0.3236209582,-1.2821968472,-0.0412898753
H,0,-2.0147926033,3.0792939674,-0.1461916907
H,0,-3.3169813975,0.983415458,-0.3195513429
C,0,2.0801454432,0.7809253048,0.1508119767
P,0,0.890096173,3.5998820386,0.0209519465
H,0,-0.1033722084,4.5964926682,0.000700526
H,0,1.269810031,3.7106698946,1.3757061025
H,0,2.3762305967,1.2784792725,1.086827343
H,0,2.3609294289,-0.2716288818,0.2773790649
C,0,2.8240505087,1.434666065,-1.0355880294
H,0,3.9041481057,1.2871380987,-0.8870107271
H,0,2.5885003645,0.863160019,-1.9473472782
Be,0,2.5919905841,3.1108507743,-1.4146976604
H,0,3.0289711531,4.0960321721,-2.2255452825

2_PH2_BeH_2

0,1
C,0,-1.4061520546,-0.094474209,0.5794506185
C,0,-0.0469369581,-0.2036780028,0.3011613112
C,0,0.6851544205,0.8801730583,-0.1940633665
C,0,0.0240889358,2.1050196562,-0.4074296756
C,0,-1.3425513328,2.2097653026,-0.1174590568
C,0,-2.0564345458,1.12036112,0.37305041
H,0,-1.9542392697,-0.9520648703,0.9570532369
H,0,0.4668217179,-1.1476781946,0.4686810111
H,0,-1.8567014237,3.1542817523,-0.2767308227
H,0,-3.1142238107,1.2220492682,0.5942292234
C,0,2.1800384406,0.7565874255,-0.3728657034
H,0,2.5095624141,1.3854583992,-1.2091877796
H,0,2.4311696257,-0.2737197242,-0.6502482388
C,0,2.9043797407,1.1856424571,0.9264263162
H,0,3.9821391859,1.2901183339,0.7444400058
H,0,2.8076367968,0.3639938371,1.6546914122
Be,0,2.2226745626,2.5398970964,1.6920392251
P,0,0.8763475673,3.5147812038,-1.2454797553
H,0,0.1243926118,4.5400832976,-0.6246115782
H,0,1.9840286657,3.670625382,-0.3667399742
H,0,1.7540488399,3.559596661,2.4105382007

2_PH2_BeF_1
0,1
C,0,-1.5460430175,-0.2909949918,0.2300994384
C,0,-0.1575433473,-0.2968146138,0.1165159863
C,0,0.5675717464,0.8863519873,-0.0486934318
C,0,-0.1522800427,2.095601224,-0.1037119586
C,0,-1.5454330541,2.1031961113,0.0081367701
C,0,-2.2458671548,0.9114682254,0.1752719695
H,0,-2.0807306212,-1.2266262064,0.3625357482
H,0,0.382892706,-1.2388102021,0.1593261098
H,0,-2.0879605704,3.0439427126,-0.0346375837
H,0,-3.327429755,0.9248265434,0.2618926119
C,0,2.0775287408,0.849996773,-0.1555676867
H,0,2.3767111632,1.3362049098,-1.0962099279
H,0,2.378220408,-0.1990898743,-0.2609450717
C,0,2.7989524755,1.5271401059,1.0321911707
H,0,3.880301844,1.3655250204,0.9136018554
H,0,2.5286301093,0.9894433794,1.9529488205
Be,0,2.5626151079,3.2208874782,1.2813951381

P,0,0.7976966819,3.6508342466,-0.2145644231
H,0,1.0998386768,3.7358068352,-1.5906327573
H,0,-0.2329219425,4.6087165976,-0.1763234907
F,0,3.0005926256,4.3428394883,2.0220272524

2_PH2_BeF_2
0,1
C,0,-1.3978722665,-0.0848676498,0.586706288
C,0,-0.0351136265,-0.1819191676,0.3231731022
C,0,0.6874894337,0.9012875098,-0.1882100007
C,0,0.0148923698,2.1138111706,-0.4303239039
C,0,-1.3564078399,2.2051081963,-0.1588058989
C,0,-2.0613886444,1.1167638053,0.3460204765
H,0,-1.939319052,-0.9404444826,0.9779205831
H,0,0.4889958101,-1.1160081264,0.5134762276
H,0,-1.8811794682,3.139272684,-0.3422767198
H,0,-3.1231776108,1.2091826026,0.5516068248
C,0,2.1833032968,0.7839910769,-0.3620480723
H,0,2.5182184047,1.4303280531,-1.1820954738
H,0,2.4369582275,-0.2399638701,-0.6597875415
C,0,2.9115472197,1.1713932106,0.9472920847
H,0,3.9916804493,1.2521141918,0.7725365256
H,0,2.7863835363,0.3475520829,1.6658435877
Be,0,2.284281929,2.55181337,1.7097461909
P,0,0.8591132526,3.5237286079,-1.2786745053
H,0,0.0776019792,4.5444107151,-0.6883576714
H,0,1.9449226885,3.7141591589,-0.3823659774
F,0,1.8516478011,3.6464066207,2.440814564

2_PH2_BeCl_1
0,1
C,0,-1.5646299577,-0.276772034,-0.2378360604
C,0,-0.1786188449,-0.3157398718,-0.1021344761
C,0,0.5767720291,0.8528628914,0.0301082457
C,0,-0.1132647605,2.080723258,0.0289528909
C,0,-1.5038935334,2.1230852017,-0.1061189958
C,0,-2.2332811189,0.944802677,-0.2400285798
H,0,-2.1220957724,-1.2022065576,-0.344346102
H,0,0.3361743864,-1.2727978842,-0.101840144
H,0,-2.0208542684,3.0790270323,-0.1082469729
H,0,-3.3122999375,0.9832867918,-0.3472718135
C,0,2.0836720327,0.7823851325,0.1653388224

P,0,0.875437778,3.6088571569,0.1031594772
H,0,-0.1031158543,4.6110922077,-0.0183353564
H,0,1.168890989,3.7511258309,1.4754701497
H,0,2.3735386144,1.2591172316,1.1139024153
H,0,2.3589335655,-0.2733619667,0.2731582977
C,0,2.8440097413,1.4509313845,-1.0036419075
H,0,3.9202507739,1.2782563258,-0.8594186084
H,0,2.592113096,0.9164440168,-1.9319947071
Be,0,2.6190407067,3.1393043654,-1.2664101482
Cl,0,3.3176831349,4.5277808203,-2.2965930369

2_PH2_BeCl_2
0,1
C,0,-1.3852096849,-0.0077399542,-0.7008751646
C,0,-0.0447862098,-0.1811191508,-0.3700196816
C,0,0.7057793727,0.8555890095,0.194886632
C,0,0.0848012824,2.0981127828,0.4246582267
C,0,-1.2634950965,2.26640884,0.0836766285
C,0,-1.9965169173,1.2243399061,-0.475887507
H,0,-1.948062339,-0.8279304118,-1.1353539511
H,0,0.4396123853,-1.138074922,-0.5515160024
H,0,-1.7468860993,3.2250919666,0.2537289104
H,0,-3.0389512454,1.3756923853,-0.7374276
C,0,2.1871819137,0.6695469534,0.4250868142
P,0,0.9571539401,3.4513862368,1.3355703831
H,0,2.1082337261,3.5749282724,0.5106718456
H,0,0.2790285443,4.5205949461,0.7031651369
H,0,2.5131322368,1.2763660682,1.2781554016
H,0,2.3907340286,-0.3722079268,0.6972718866
C,0,2.9753964092,1.0785125297,-0.8441228458
H,0,4.0423014127,1.1930434294,-0.6162435841
H,0,2.9135554617,0.2505204082,-1.5675024789
Be,0,2.3181382198,2.4163672922,-1.6474083331
Cl,0,1.7241575589,3.7754306089,-2.6912596971

2_COOH_BeH_1
0,1
C,0,-1.4815570621,-0.2456026319,-0.3004282111
C,0,-0.1057111708,-0.2715189261,-0.0969881395
C,0,0.645298122,0.9019366515,0.0296891113
C,0,-0.05921321,2.12101804,-0.0639784104
C,0,-1.452611088,2.1521540175,-0.2306369227

C,0,-2.1646070805,0.9696329619,-0.3624341711
H,0,-2.027324872,-1.1788209278,-0.401926442
H,0,0.4115618142,-1.2241390824,-0.02677536
H,0,-1.9610991941,3.1096535157,-0.2714198166
H,0,-3.2387357972,0.9929559881,-0.5100570366
C,0,2.1319326739,0.8142249576,0.2678074863
C,0,0.6458601187,3.4099273188,0.0211237791
O,0,1.7884439265,3.6559214751,-0.3819336682
O,0,-0.0555572304,4.3919758736,0.5693956911
H,0,0.4684534857,5.2078759845,0.5100659472
H,0,2.4059901465,1.5041528811,1.0774400598
H,0,2.3386303958,-0.1953684377,0.6458311217
C,0,2.9401018038,1.1570157279,-1.0005151001
H,0,4.0017815079,0.947306805,-0.802717223
H,0,2.6567326261,0.463054084,-1.8056783464
Be,0,2.7426807238,2.8079699229,-1.4890387152
H,0,3.11540165,3.6204283707,-2.5130971037

2_COOH_BeH_2
0,1
C,0,-1.4436760084,-0.4871460087,-0.3521160097
C,0,-0.065960311,-0.3437210879,-0.2326368106
C,0,0.5387515628,0.9017401304,-0.0224174212
C,0,-0.3169514042,2.0169742061,0.0720965245
C,0,-1.7063168435,1.8791871944,-0.0795265903
C,0,-2.2740935951,0.6313048991,-0.2814318363
H,0,-1.8706434394,-1.4728507241,-0.5103231865
H,0,0.5742081037,-1.2180783803,-0.3099620062
H,0,-2.3235848668,2.7701703236,-0.0283186956
H,0,-3.3490640285,0.5311097412,-0.3843518185
C,0,2.0422230853,0.9604105808,0.0922255407
H,0,2.4221241995,1.8027809758,-0.500579056
H,0,2.4356396991,0.0544615274,-0.3846077275
C,0,2.4972561725,1.0899223059,1.5628710572
H,0,2.014833628,0.3025073032,2.1582042334
C,0,0.1158108588,3.4141627867,0.2951464426
O,0,-0.4815753304,4.4033080291,-0.0257940123
H,0,3.5740201863,0.8718131117,1.616244564
Be,0,2.2597558128,2.6848696751,2.1477488181
O,0,1.3042752141,3.5914239282,1.0108315861
H,0,1.3990904229,4.5464318332,1.1740745614

2_COOH_BeF_1
0,1
C,0,-1.4762270378,-0.2426501614,-0.3004617705
C,0,-0.0992480207,-0.2637643003,-0.1033840183
C,0,0.6484844494,0.9108683708,0.0275405606
C,0,-0.0616782147,2.1281374089,-0.0529874416
C,0,-1.4556635869,2.1538062181,-0.2147570419
C,0,-2.1641975764,0.9699202508,-0.3515047368
H,0,-2.0175085878,-1.1780191183,-0.40530395
H,0,0.4216853408,-1.2149843024,-0.0417900427
H,0,-1.9688862769,3.1088879913,-0.2486199407
H,0,-3.2388309087,0.9904320558,-0.4960601185
C,0,2.1367595414,0.8200296491,0.2595635294
C,0,0.6318278227,3.424492376,0.0249206791
O,0,1.7698910027,3.6806277574,-0.383449908
O,0,-0.0771058913,4.4020215018,0.5680841235
H,0,0.4371130261,5.2236853421,0.4987221664
H,0,2.415171363,1.5038209825,1.0728977593
H,0,2.3428525933,-0.192077726,0.6300208852
C,0,2.9454911126,1.1646264232,-1.0080870204
H,0,4.0039993147,0.9384107302,-0.8168603218
H,0,2.6463492398,0.4862477527,-1.8197966722
Be,0,2.7729512385,2.829870884,-1.4488725849
F,0,3.2503248263,3.7131553537,-2.4607108754

2_COOH_BeF_2
0,1
C,0,-1.4401344879,-0.4904389511,-0.3599983168
C,0,-0.0647032898,-0.3424732631,-0.2211328637
C,0,0.5347710433,0.9049265075,-0.0095223014
C,0,-0.3251239375,2.0195824035,0.064075191
C,0,-1.7113102366,1.8766591326,-0.1064362928
C,0,-2.2737443011,0.6263752113,-0.3091745931
H,0,-1.8617122813,-1.4784485507,-0.5178524509
H,0,0.5782432881,-1.2159751861,-0.2840073763
H,0,-2.3315675743,2.7661815745,-0.0688409401
H,0,-3.3469710083,0.5231053463,-0.4270658506
C,0,2.0383592573,0.9602562852,0.1222586209
H,0,2.4276841643,1.8040219374,-0.4617956938
H,0,2.43379949,0.0556167245,-0.35455276
C,0,2.4862089025,1.0768417092,1.5972326367
H,0,1.9976452,0.2887693895,2.1845825005

C,0,0.0973379318,3.4195875133,0.2961677772
O,0,-0.4982535614,4.4069303396,-0.0320363994
H,0,3.5626028352,0.8630410468,1.6560420898
Be,0,2.2265318987,2.6728424425,2.1612374065
O,0,1.2713054642,3.5914055563,1.0304651949
H,0,1.3683390818,4.5426171454,1.2226592978
F,0,2.6384141208,3.6696946856,3.0933471239

2_COOH_BeCl_1
0,1
C,0,-1.4746249846,-0.2331958362,-0.3644208969
C,0,-0.102481583,-0.2627514195,-0.1368315849
C,0,0.6450894953,0.9066964998,0.0337945715
C,0,-0.060892293,2.127559292,-0.0395577932
C,0,-1.4514121036,2.1608539008,-0.2318257073
C,0,-2.1589454684,0.9819323515,-0.4072324695
H,0,-2.0159380362,-1.1648862035,-0.4988338489
H,0,0.414050197,-1.2166822489,-0.0816047276
H,0,-1.9608604078,3.118201169,-0.2590079051
H,0,-3.2301523028,1.0074239294,-0.5737119161
C,0,2.127265784,0.8088183529,0.3007597772
C,0,0.6314867947,3.4184266657,0.0828164605
O,0,1.7855582533,3.678156183,-0.2878540923
O,0,-0.0836432407,4.3896333783,0.6221119828
H,0,0.4350170067,5.2107540493,0.5799470321
H,0,2.3841662281,1.4679401047,1.1409831773
H,0,2.3257625178,-0.2137423928,0.6451394239
C,0,2.9690366657,1.1916535554,-0.9344312553
H,0,4.0257448693,0.9847740539,-0.7140668255
H,0,2.7109728738,0.5255601493,-1.769682561
Be,0,2.7627663572,2.850373176,-1.3482795308
Cl,0,3.3570596373,3.9799547999,-2.7333807608

2_COOH_BeCl_2
0,1
C,0,-1.5045696625,-0.2640003974,-0.3918039689
C,0,-0.123587905,-0.2639558765,-0.2286225969
C,0,0.5879986985,0.8957015828,0.0995267502
C,0,-0.1604746915,2.079709176,0.2536570815
C,0,-1.5585051018,2.0762432854,0.1210751707
C,0,-2.2325481154,0.9116189022,-0.2107283784
H,0,-2.0146670414,-1.1867707138,-0.6508052974

H,0,0.4317795654,-1.1897436785,-0.3500304515
H,0,-2.0971692426,3.0052535946,0.2766734845
H,0,-3.3108463422,0.9190768411,-0.3261109646
C,0,2.0854337107,0.7994130915,0.2603331859
C,0,0.393297798,3.4088920361,0.5915527081
O,0,-0.1754191505,4.2997977386,1.1534282352
O,0,1.6972156652,3.6769240167,0.1388823452
H,0,1.8802022226,4.6193508701,0.3167593932
H,0,2.3968577732,1.3549840425,1.1544475893
H,0,2.3218129037,-0.2521349473,0.4611686751
C,0,2.8270176374,1.330316157,-0.9865695361
H,0,3.8838737563,1.0353288232,-0.9233430973
H,0,2.4352685908,0.8279784866,-1.8806025961
Be,0,2.7585644419,3.0342549007,-1.0378748046
Cl,0,3.6616940192,4.5123001884,-1.7666679272

2_CONH2_BeH_1
0,1
C,0,-3.0723719106,5.1378852445,-0.4530849073
C,0,-2.9220301846,4.3870706333,0.7092069009
C,0,-1.6799103376,3.8805339669,1.1048067547
C,0,-0.5782759108,4.1656097296,0.2760150831
C,0,-0.7204439145,4.9333395365,-0.8869219546
C,0,-1.9695911012,5.415116737,-1.2593622792
H,0,-4.054357957,5.5071147805,-0.7327622021
H,0,-3.7861918609,4.1807876902,1.3347572298
H,0,0.1435172285,5.1196715716,-1.5198288953
H,0,-2.0819229954,5.9930707484,-2.1704233154
C,0,-1.5609694398,3.0428831003,2.3528194802
C,0,0.774980998,3.6615878376,0.6330447236
O,0,1.0175899038,2.4889877563,0.9829190196
N,0,1.793552074,4.5275079378,0.5388717954
H,0,1.6295249411,5.5067372846,0.3682683965
H,0,2.7269294853,4.2016398103,0.7473325942
H,0,-0.6784011431,3.3667706726,2.9221036472
H,0,-2.4310191378,3.2657722505,2.9849481397
C,0,-1.4310125115,1.5462057363,2.0019314344
H,0,-1.4506889814,0.9654032466,2.9364170135
H,0,-2.330213472,1.231265094,1.4510676696
Be,0,0.0150977825,1.1712559197,1.1001235533
H,0,0.529432165,0.0652174048,0.4931143283

2_CONH2_BeH_2
0,1
C,0,-1.5739289563,-0.2701133238,-0.0464076784
C,0,-0.1854562284,-0.2838245847,0.0196626189
C,0,0.5792988665,0.8895924195,0.0738602597
C,0,-0.129871103,2.1130316042,0.0431344101
C,0,-1.5316502151,2.1229898403,-0.0372350285
C,0,-2.25796321,0.9433180439,-0.0744911567
H,0,-2.1199911331,-1.2079053536,-0.0836180267
H,0,0.3352992616,-1.2369139628,0.022861766
H,0,-2.0324779191,3.0848236236,-0.0693139062
H,0,-3.3409677155,0.9701742381,-0.1305191619
C,0,2.08884272,0.7321534215,0.0976385738
H,0,2.2851836776,-0.3463320511,0.0992212461
C,0,0.4673475397,3.4790354808,0.0806024755
O,0,-0.1262521982,4.4809642365,-0.2393760909
N,0,1.8363678367,3.6187035561,0.5023223876
H,0,2.0017582817,4.5935642658,0.7464134226
H,0,2.0782253423,2.9923752663,1.2692518592
H,0,2.481593041,1.0735743657,1.0700046072
C,0,2.8167253638,1.4460574253,-1.0613805689
H,0,3.8436412134,1.0553081636,-1.1142096391
H,0,2.3512657823,1.1330869355,-2.0070835117
Be,0,2.9106484518,3.1634931236,-0.9052728661
H,0,3.5497688103,4.2493644056,-1.4020210315

2_CONH2_BeF_1
0,1
C,0,-1.4370697684,-0.4095649537,-0.3168730294
C,0,-0.0512726193,-0.3461451729,-0.2015766234
C,0,0.6232143691,0.8703824141,-0.0577915679
C,0,-0.1613517864,2.0393890441,-0.0378057412
C,0,-1.555205653,1.9828688817,-0.1672885579
C,0,-2.197952857,0.7581784576,-0.2993246709
H,0,-1.9255442045,-1.3736666711,-0.4192237483
H,0,0.5350147225,-1.2606870996,-0.2224460673
H,0,-2.1390784248,2.898716314,-0.124626792
H,0,-3.2787631471,0.7156574904,-0.3802439705
C,0,2.1237194302,0.8925081796,0.0943743647
H,0,2.5391526641,1.6888158944,-0.5388019415
H,0,2.510197487,-0.0521648387,-0.3101938221
C,0,2.5312193666,1.131876129,1.5630660812

H,0,2.1227423375,0.3197480248,2.1821147434
C,0,0.4766913702,3.3749651251,0.11642845
O,0,1.342832493,3.6450827894,0.9732229192
N,0,0.0607908912,4.3477758216,-0.7037487379
H,0,0.4506315371,5.2736688817,-0.5937410574
H,0,-0.5599753131,4.1513097717,-1.4726795165
H,0,3.6235875602,1.0350131876,1.642359024
Be,0,2.0262271595,2.6923919487,2.1509613842
F,0,2.0946779155,3.3710387405,3.4072827975

2_CONH2_BeF_2
0,1
C,0,-1.4221154923,-0.1824477329,-0.975746192
C,0,-0.2357043147,-0.3032680526,-0.2611961725
C,0,0.3492465582,0.7723952158,0.4213344937
C,0,-0.3207477943,2.0161486571,0.3394333932
C,0,-1.5128564473,2.1365808979,-0.3933525872
C,0,-2.071726076,1.0479153039,-1.0436737514
H,0,-1.8346493215,-1.049271755,-1.4830365434
H,0,0.2670936556,-1.2654060931,-0.2301793218
H,0,-1.9858802718,3.1118752443,-0.4374184
H,0,-2.9963410447,1.158639818,-1.5998983758
C,0,1.6644775386,0.5006328874,1.131334317
H,0,1.8702906786,-0.5670383311,0.9942797801
H,0,1.525959082,0.6074876289,2.2195480498
C,0,2.8456351133,1.3710248459,0.6489777687
H,0,3.7770083379,0.9197684592,1.0194472163
C,0,0.1183593496,3.300508804,0.9585255202
O,0,-0.3051561931,4.3823750929,0.6303138998
N,0,1.1245269218,3.2572760423,1.9875315302
H,0,1.1453201949,4.1609751536,2.4605152519
H,0,0.9784121954,2.4944114641,2.6470673834
H,0,2.9082214125,1.2957855677,-0.445147328
Be,0,2.7668167969,2.9955313731,1.2201243149
F,0,3.5571528606,4.1647013784,1.404119893

2_CONH2_BeCl_1
0,1
C,0,-1.8036230736,-0.1884759315,-0.6870046084
C,0,-0.4585749702,-0.282501872,-1.0322961781
C,0,0.503614834,0.5777641139,-0.4947714462
C,0,0.0526923484,1.5552558856,0.413564098

C,0,-1.3020686914,1.6629785626,0.75505179
C,0,-2.2327410182,0.7863430676,0.2122791783
H,0,-2.5199390823,-0.8796489727,-1.1204263676
H,0,-0.1341889184,-1.0421433187,-1.7380812469
H,0,-1.6190619296,2.4087452513,1.4795496384
H,0,-3.2777876255,0.8566928756,0.4940477481
C,0,1.9562114632,0.419542883,-0.8685927599
H,0,1.9969857275,-0.1349590433,-1.8149175384
C,0,1.0107548782,2.5066366549,1.035040572
O,0,2.1057114149,2.1729874394,1.5407794749
N,0,0.6584233881,3.7945717174,1.0802203502
H,0,1.2751477293,4.4498591379,1.5407645492
H,0,-0.1631376693,4.1277126049,0.6012596295
H,0,2.3875930949,1.4088135704,-1.0752112143
C,0,2.7481780595,-0.2658591161,0.2650845711
H,0,2.3220854334,-1.2629121589,0.4479611897
H,0,3.7746569103,-0.4488066491,-0.0832046521
Be,0,2.7615358201,0.6841664104,1.7129508011
Cl,0,3.4049658767,0.3678278873,3.4613984213

2_CONH2_BeCl_2
0,1
C,0,-1.5657139095,-0.2641492216,-0.1054480781
C,0,-0.1779183146,-0.2737092741,-0.0239509523
C,0,0.5814784,0.8999256041,0.0755253338
C,0,-0.1334597512,2.1214317027,0.0754933994
C,0,-1.5353413653,2.1265149007,-0.0166864964
C,0,-2.255631446,0.9461491304,-0.1007459085
H,0,-2.1058780994,-1.2034257654,-0.1762820197
H,0,0.3464552534,-1.2244787681,-0.0429821299
H,0,-2.0411439918,3.086080163,-0.0216707616
H,0,-3.3380287076,0.9693273187,-0.1658693715
C,0,2.0909477535,0.7411419572,0.1191164765
H,0,2.2872865352,-0.3368832695,0.1177886437
C,0,0.4495633718,3.491005696,0.1540056494
O,0,-0.1604609448,4.4998859955,-0.0996109507
N,0,1.8401474354,3.633226481,0.5339221215
H,0,2.0025147297,4.6134945848,0.768752213
H,0,2.0918814415,3.0237296686,1.3124001803
H,0,2.4711295552,1.0794376275,1.0970247958
C,0,2.8376165793,1.4546385455,-1.0287162422
H,0,3.8708521567,1.0803952262,-1.0539404359

H,0,2.3964766266,1.1362925036,-1.9830983582
Be,0,2.8841469077,3.1606315415,-0.8501289995
Cl,0,3.7803147841,4.6409116518,-1.5695881086
